# ^177^Lu-Labeled Magnetic Nano-Formulations: Synthesis, Radio- and Physico-Chemical Characterization, Biological Applications, Current Challenges, and Future Perspectives

**DOI:** 10.3390/molecules30214290

**Published:** 2025-11-04

**Authors:** Eleftherios Halevas, Despoina Varna

**Affiliations:** 1 Institute of Biosciences & Applications, National Centre for Scientific Research “Demokritos”, 15310 Athens, Greece; 2 Laboratory of Biochemistry, Department of Chemistry, Aristotle University of Thessaloniki, 54124 Thessaloniki, Greece; 3 Laboratory of Inorganic Chemistry, Department of Chemistry, Aristotle University of Thessaloniki, 54124 Thessaloniki, Greece; varndesp@chem.auth.gr

**Keywords:** Lutetium-177, magnetic nano-formulations, radiolabeling, imaging, targeted radiation therapy, theranostics, nanomedicine

## Abstract

The advent of nanotechnology has revolutionized the field of medicine, particularly in the development of targeted therapeutic strategies. Among these, radiolabeled nanomaterials have emerged as promising tools for both diagnostic and therapeutic applications, offering precise delivery of radiation to diseased tissues while minimizing damage to healthy ones. Notably, Lutetium-177 (^177^Lu) has gained significant attention due to its favorable emission properties and availability that render it suitable for imaging and therapeutic purposes. When integrated with magnetic nano-formulations, ^177^Lu-labeled systems combine the benefits of targeted radiation therapy (TRT) with the unique properties of magnetic nanoparticles (MNPs), such as magnetic resonance imaging (MRI) contrast enhancement and magnetically guided drug delivery to address challenges in diagnosis and treatment of diseases, such as cancer. By examining the latest advancements in their design, particularly surface functionalization and bioconjugation strategies, this study aims to highlight their efficacy in targeted therapy, imaging, and theranostic applications. Furthermore, we discuss the current challenges, such as scalability, biocompatibility, and regulatory hurdles, while proposing future directions to enhance their clinical translation. This comprehensive review underscores the transformative potential of ^177^Lu-labeled magnetic nano-formulations in precision medicine and their role in shaping the future of therapeutic interventions.

## 1. Introduction

Cancer remains a major global health threat, standing as the second most common cause of mortality worldwide with an estimated 10 million fatalities recorded in 2022 alone [1]. The World Health Organization projects a 77% increase in cancer cases by 2050, underscoring the pressing need for more effective treatment modalities [2]. While conventional approaches, including surgical resection, cytotoxic chemotherapy, and external beam radiation therapy, remain cornerstones of oncologic care, their clinical utility is often limited by significant drawbacks. These include non-specific biodistribution of chemotherapeutic agents leading to systemic toxicity, radiation damage to healthy tissues adjacent to tumors, and the emergence of multidrug resistance mechanisms in malignant cells [3].

In this therapeutic landscape, TRT has emerged as a paradigm-shifting approach that combines the precision of molecular targeting with the potent cytotoxic effects of ionizing radiation [4]. This innovative strategy employs radiopharmaceuticals designed to selectively accumulate in tumor tissue through various mechanisms, including receptor-ligand interactions, antigen–antibody binding, or metabolic trapping [5]. The fundamental advantage of TRT lies in its ability to deliver lethal radiation doses specifically to cancer cells while largely sparing normal tissues, thereby achieving an improved therapeutic index compared to conventional treatments [6].

^177^Lu has emerged as one of the most clinically valuable radionuclides for TRT and Single Photon Emission Computed Tomography (SPECT) imaging due to its ideal physico-chemical and emission properties. These properties facilitate the ‘crossfire’ effect on neighboring cancer cells while sparing the normal surrounding tissue with minimal effect [7]. The versatility of ^177^Lu, combined with its well-characterized chemistry for stable chelation with various polydentate macrocyclic ligands (e.g., tetraxetan), makes it a cornerstone of modern TRT development, with ongoing research focused on optimizing dosimetry, combination therapies, and personalized treatment approaches [8,9]. The success of ^177^Lu-labeled compounds, particularly [^177^Lu]Lu-DOTATATE (Lutathera^®^) for neuroendocrine tumors [10] and [^177^Lu]Lu-Vivipotide tetraxetan (Pluvicto^®^) for the treatment of prostate tumors expressing somatostatin [11], has demonstrated the clinical potential of this radionuclide in peptide receptor radionuclide therapy (PRRT) and radioligand therapy, showing significant improvements in progression-free survival with favorable safety profiles [12].

Recent advances in nanotechnology have further enhanced the potential of ^177^Lu-based therapies by incorporating MNPs as multifunctional carriers. These nano-formulations combine the benefits of radiation therapy with magnetic targeting, enabling improved tumor accumulation and retention [13]. MNPs, particularly those based on iron oxides (e.g., Fe_3_O_4_), exhibit excellent biocompatibility, superparamagnetic behavior, and surface functionalization versatility, rendering them ideal for theranostic applications [14]. When labeled with ^177^Lu, these NPs can be guided to tumor sites using external magnetic fields, enhancing therapeutic precision while reducing off-target effects [15].

The synthesis of ^177^Lu-labeled magnetic nano-formulations involves critical steps, including NP preparation, surface modification, radiolabeling optimization, and thorough physico-chemical characterization [16]. Key parameters such as particle size, surface charge, colloidal stability, radiolabeling efficiency, and *in vitro*/*in vivo* behavior must be meticulously evaluated to ensure therapeutic efficacy [17]. Additionally, the integration of targeting ligands (e.g., peptides, antibodies, or small molecules) can further enhance tumor specificity, paving the way for personalized cancer therapy [18].

This review article provides, for the first time, a comprehensive analysis of ^177^Lu-labeled magnetic nano-formulations, consolidating current knowledge on their synthesis strategies, radiochemical and physico-chemical properties, and biological applications. Furthermore, we discuss current challenges, such as scalability, long-term stability, functional implications, and potential toxicity, while exploring future perspectives, including combination therapies, multimodal imaging, and smart stimulus-responsive designs. By critically evaluating recent advancements, this review aims to highlight the transformative potential of ^177^Lu-labeled magnetic nano-formulations in oncology and inspire further research toward clinical translation.

## 2. ^177^Lutetium

### 2.1. ^177^Lutetium Chemistry

Lutetium (Lu), the final element in the lanthanide series, possesses 71 electrons with an electronic configuration of [Xe]4f^14^5d^1^6s^2^. In chemical reactions, Lu typically loses its two 6s electrons and the single 5d electron, forming a trivalent (+3) cation. This +3 oxidation state is dominant across lutetium’s compounds, including oxides and halides, aligning it with conventional lanthanide behavior. Among the lanthanides, Lu(III) has the smallest atomic radius due to lanthanide contraction, contributing to its exceptional hardness and density. While most lanthanides belong to the f-block, lutetium’s filled 4f orbital (14 electrons) allows it to also be classified as the first d-block element in the sixth period. In the +3 state, Lu(III) retains empty s, p, and d orbitals while maintaining a closed 4f shell. The tightly bound f-electrons, shielded by a highly effective nuclear charge, do not participate in bonding, making Lu(III) a hard Lewis acid whose chemistry is primarily dictated by its vacant s, p, and d orbitals. With an ionic radius of just 86.1 pm, the smallest of all lanthanides, Lu(III), accommodates fewer ligands due to spatial constraints. Coordination geometry is largely determined by ligand-ligand repulsions rather than directional bonding contributions from s, p, or d orbitals. Lu(III) complexes exhibit variable coordination numbers, typically ranging from 6 to 9, demonstrating the element’s versatile bonding characteristics [19].

The International Commission on Radiological Protection (ICRP) has catalogued over 1200 radionuclides, yet only a few dozen are routinely used in clinical and scientific settings [20]. As shown in Table 1, these radionuclides possess distinct physical and biochemical properties, including half-life, decay mode, radiation energy, and particle range, which determine their specific applications. The table also indicates their common production methods and reactions. For clinical purposes, radionuclides are broadly categorized as either imaging isotopes or therapeutic isotopes, which correspond to their primary decay modes (e.g., γ-emitters for imaging and α−, β−, or Auger electron emitters for therapy) [21,22].

The selection of an optimal radionuclide is a multifactorial decision, extending beyond the general category of radiation. Critical considerations involve: (i) ensuring the emission range is appropriate for the dimensions of the target structures, with longer-range emitters potentially compensating for heterogeneous uptake of the carrier molecule, (ii) aligning the radionuclide’s physical half-life with the carrier’s pharmacokinetic profile to maximize energy deposition within the target at the critical time, (iii) accounting for the abundance of photon emissions, which is relevant for both diagnostic imaging and radiation protection, and (iv) evaluating the implications of any radioactive daughter nuclides, which may contribute to the overall dose but also pose a risk of redistributing away from the intended site [23].

**Table 1:** Radionuclide properties. Data from [24,25].

**Radionuclides for SPECT Imaging**					
**Radionuclide**	**Haf-Life**	**Max Energy (keV)**	**Decay**	**Production**	**Common Production Reaction**
Au-198	2.7 days	960	β−, γ	Cyclotron	^197^Au(n,g)^198^Au
Au-199	3.1 days	452.6	β−, γ	Cyclotron	^198^Au(n,g)^199^Au
Co-57	270 days	692	Electron capture, γ	Cyclotron	^56^Fe(d,n)^57^Co
Fe-59	44.5 days	1291	β−, γ	Cyclotron	^59^Co(p,n)^59^Fe
Ga-67	78.3 h	300	Auger e^−^, γ	Cyclotron	^68^Zn(p,2n)^67^Ga
Gd-153	240.4 days	103	Electron capture, γ	Cyclotron	^152^Gd(n,g)^153^Gd
In-111	2.81 days	245	γ	Cyclotron	^111^Cd(p,n)^111^In
I-123	13.3 h	159	Auger e^−^, γ	Cyclotron	^127^I(p,5n)^123^Xe
Re-186	91 h	1080	β−, γ	Cyclotron	^186^W(p,n)^186^Re
Tc-99m	6.0 h	140	γ	Generator	^99^Mo/^99m^Tc
Tl-201	3.0 days	71	γ	Cyclotron	^203^Tl(p,3n)^201^Pb
**Radionuclides for PET imaging**					
As-72	25.9 h	3320	β+	Cyclotron	^72^Ge(p,n)^72^As
Br-76	16 h	3980	β+	Cyclotron	^76^Se(p,n)^76^Br
C-11	20.4 min	961	β+	Cyclotron	^14^N(p,a)^11^C
Cu-62	9.7 min	2926	β+	Generator	^62^Zn/^62^Cu
Cu-64	12.7 h	656	Electron capture, β+, β−	Cyclotron	^64^Ni(p,n)^64^Cu
F-18	109.7 min	634	Electron capture, β+	Cyclotron	^18^F(F^−^):^18^O(p,n)^1^8F
Ga-68	67.6 min	1899	Electron capture, β+	Generator/ Cyclotron	^68^Ge/^68^Ga
Ge-69	39.1 h	1205	β+	Cyclotron	^69^Ga(p,n)^69^Ge
I-124	4.2 days	2100	Electron capture, β+	Cyclotron	^124^Te(p,n)^124^I
Mn-52	5.6 days	1434	β+	Cyclotron	^52^Cr(p,n)^52^Mn
N-13	9.9 min	1199	β+	Cyclotron	^16^O(p,a)^13^N
O-15	2.1 min	1732	β+	Cyclotron	^15^N(p,n)^15^O
Rb-82	1.3 min	3378	Electron capture, β+	Generator	^82^Sr/^82^Rb
Y-86	14.7 h	3150	β+	Cyclotron	^86^Sr(p,n)^86^Y
Zr-89	78.4 h	900	Electron capture, β+	Cyclotron	^89^Y(p,n)^89^Zr
**Radionuclides for therapy applications (β-emission, Linear energy transfer ~0.2 keV^.^μm^−1^)**					
**Radionuclide**	**Haf-life**	**Maximum energy (keV)**	**Decay**	**Production**	**Maximum particle range**
Au-198	2.7 days	960	β−, γ	Cyclotron	4.0 mm
Y-90	64.0 h	2280	β−	Generator	12.0 mm
Lu-177	6.7 days	500	β−, γ	Cyclotron	1.5 mm
I-131	8.0 days	610	β−, γ	Fission	2.0 mm
Cu-67	62 h	577	β−, γ	Cyclotron	1.8 mm
Re-186	91 h	1080	β−, γ	Cyclotron	5.0 mm
Re-188	16.9 h	2120	β−, γ	Generator	10.0 mm
**Radionuclides for therapy applications (α-emission, Linear energy transfer ~80 keV^.^μm^−1^)**					
At-211	7.2 h	6000	α	Cyclotron	0.08 mm
Ac-225	10 days	8000	α, β−	Cyclotron	0.1 mm
Bi-212	60.6 min	6000	α, β−	Cyclotron	0.09 mm
Bi-213	46 min	6000	α, β−	Cyclotron	<0.1 mm
Ra-223	11.4 days	7000	α, β−	Cyclotron	<0.1 mm
Pb-212	10.6 h	7800	α, β−	Cyclotron	<0.1 mm
Tb-149	4.2 h	400	α	Cyclotron	<0.1 mm
**Radionuclides for therapy applications (Auger-emission, Linear energy transfer ~4–26 keV^.^μm^−1^)**					
Ga-67	78.3 h	300	Auger e^−^, γ	Cyclotron	10 nm
I-123	13.3 h	159	Auger e^−^, γ	Cyclotron	10 nm
I-125	60.5 days	27	Auger e^−^, γ	Neutron activation	10 nm

The two main forms of radionuclide imaging are SPECT and Positron Emission Tomography (PET), relying on single-photon and positron-emitting radionuclides [26]. The short half-lives of these isotopes help limit radiation dose to patients. In contrast, choosing a radionuclide for therapy requires different criteria. An ideal therapeutic agent has a half-life between 6 h and 10 days [27] and emits high linear energy transfer radiation for potent cell killing [28]. These requirements are met by three primary types of radionuclides: β-emitters, α-emitters, and Auger electron emitters. The basic characteristics of these types of particles are presented in Table 2 [29].

**Figure 1:**
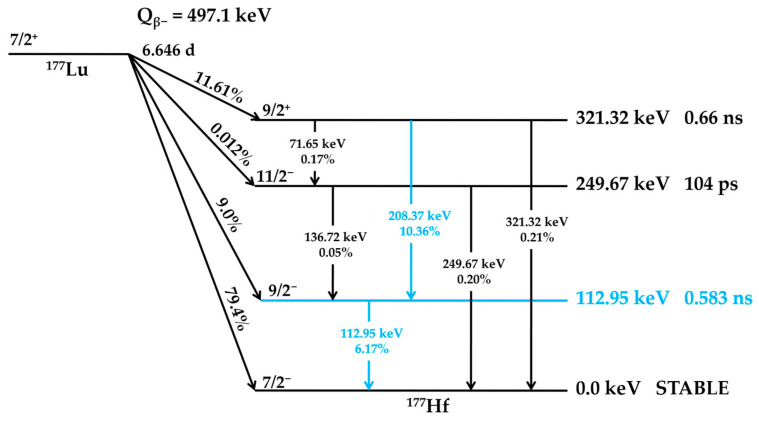
Simplified decay scheme of ^177^Lu. Levels, spins, parities, energies, and half-lives of the excited states are based on literature-reported data [34,35,36]. The decay pathways and energy states involved in gamma-ray cascades are highlighted in blue.

^177^Lu undergoes radioactive decay with a half-life of 6.646 days. In 79.4% of decay events, it emits beta particles (β−) with a maximum energy (E_βmax_) of 497.1 keV, transitioning directly to the stable ground state of Hafnium-177 (^177^Hf). Additionally, 9.0% of decays occur via β− emission with E_βmax_ = 385 keV, while 11.61% involve β− emission at E_βmax_ = 177 keV, both leading to excited states of ^177^Hf at 249.67 keV and 321.32 keV above the ground state, respectively. These excited states then relax to the ground state by emitting gamma photons. The emitted gamma rays of 112.95 keV and 208.37 keV occur via an intermediate state that has a half-life of 0.583 nanoseconds [30]. A simplified decay scheme of ^177^Lu is presented below in Figure 1. The energy of beta particles enables optimal soft tissue penetration up to 2 mm, effectively destroying tumor cells while minimizing radiation exposure effects on surrounding healthy tissues [31,32,33].

In recent years, the radionuclide ^177^Lu has gained significant interest across research, commercial, and clinical fields due to its potential in various diagnostic and therapeutic applications [37,38,39,40,41,42,43,44]. Although it emerged relatively late, in a short period, ^177^Lu has not only proven its effectiveness but also secured a prominent position in nearly all areas of *in vivo* radionuclide imaging and treatment, becoming a leading choice for TRT. ^177^Lu is ideal for radiolabeling bioactive tracer molecules, using a chemistry similar to the positron emitter ^68^Ga. This compatibility enables the creation of a matched diagnostic/therapeutic pair. Such a pair facilitates a theranostic strategy, where high-resolution quantitative PET imaging with ^68^Ga is used for patient selection, followed by targeted therapy with ^177^Lu for treatment and monitoring. Consequently, these properties establish ^177^Lu as an excellent isotope for image-guided radionuclide therapy [45].

**Figure 2:**
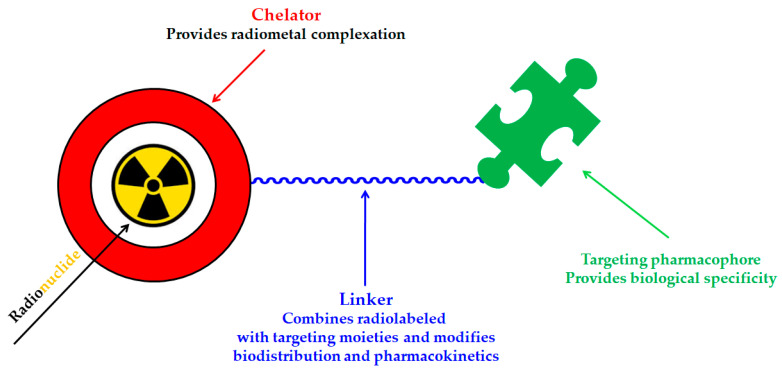
Schematic representation of the key components of a bifunctional radioligand.

A key benefit of ^177^Lu-based therapies is their favorable safety profile. Unlike conventional chemotherapy, which often harms healthy tissues, ^177^Lu bifunctional radioligand treatments selectively target cancer cells, reducing damage to normal tissues. As a result, patients typically experience fewer side effects and an improved quality of life. This approach is particularly valuable for advanced-stage cancer patients with limited conventional treatment options [46]. A bifunctional radioligand (Figure 2) consists of three key components: a chelator that binds the radiometal and a pharmacophore that provides biological specificity to the final radiolabeled conjugate complex. To facilitate conjugation, chelators are functionalized with reactive groups that allow covalent attachment to biomolecules like antibodies, peptides, drugs, nucleotides, human *Escherichia coli* enterotoxins, or even bone pain palliation agents, particulates, steroids, porphyrins, nitroimidazoles, fullerenes, and various types of NPs [19]. The pharmacokinetic properties of the conjugate can be adjusted by introducing a linker between the chelator and the targeting vector. Typically composed of hydrocarbon chains (CH_2_)_n_, polyethylene glycol, or peptide sequences, these linkers affect biodistribution and pharmacokinetics by modifying charge and hydrophilicity [47,48,49,50]. Indicative examples of current ^177^Lu-labeled bifunctional radioligand-based radiopharmaceuticals and their treatment conditions are presented in Table 3.

^177^Lu bifunctional radioligand therapies are based on highly efficient and selective chemical reactions that involve binding ^177^Lu to a targeting molecule via a “click” reaction. In general, the concept of “click chemistry” describes a class of efficient and reliable chemical reactions ideal for creating functional materials and bioconjugates. These ligation processes typically join two molecular components under mild conditions, yielding the desired product rapidly and with high efficiency. Significant advancements over the past twenty years have broadened the scope of click reactions, with many now proceeding effectively in water without sacrificing speed or selectivity. A key advantage is the straightforward purification of the final product, as these reactions often allow for the simple removal of unreacted starting materials and byproducts, eliminating the need for complex separation techniques [52].

**Figure 3:**
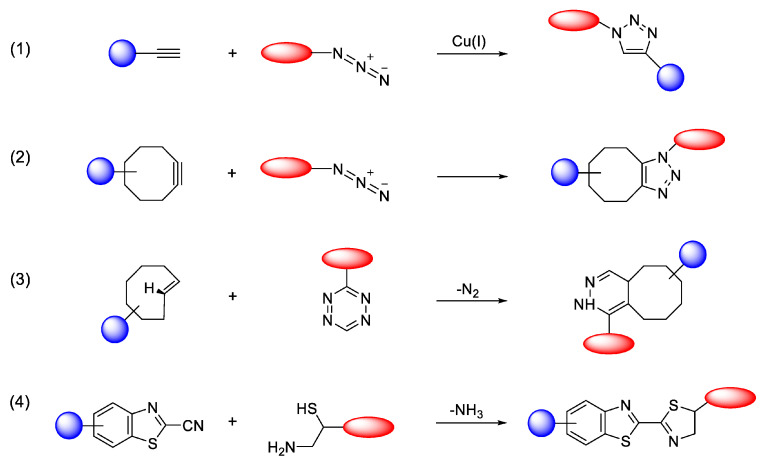
Selected bioorthogonal conjugation reactions. (**1**) CuAAC; (**2**) SPAAC; (**3**) IEDDA; (**4**) condensation reaction between 2-cyanobenzothiazole (CBT) and 1,2-aminothiol (*N*-terminal cysteine). Adopted from Mushtaq, S.; Yun, S.-J.; Jeon, Molecules; published by MDPI, 2019 [52].

Consequently, click chemistry has become a valuable tool across diverse fields, including biochemistry, materials science, drug development, and the synthesis of radiolabeled compounds. While other ligation methods like thiol-Michael additions [53] or oxime formation exist [54], they often suffer from limitations such as low specificity or instability in aqueous environments. A major breakthrough came with the introduction of the copper(I)-catalyzed azide-alkyne cycloaddition (CuAAC) (Figure 3) [55] as a powerful click reaction for bioconjugation. Its success is largely due to the bioorthogonality of azide and alkyne functional groups, which do not react with native biomolecules [52].

However, concerns about the cytotoxicity of the required copper catalyst have prompted the development of metal-free alternatives. Reactions such as the strain-promoted azide-alkyne cycloaddition (SPAAC) [56] and the inverse-electron-demand Diels–Alder (IEDDA) [57] (Figure 3) have since emerged as highly useful, biocompatible click reactions and are now widely adopted in biological research [52].

The principles of click chemistry are increasingly being adopted for synthesizing radioisotope-labeled compounds used in nuclear medicine for both diagnostic imaging (PET and SPECT) and therapy. This application is particularly vital for short-lived isotopes. Metal-free click reactions are exceptionally well-suited for this purpose, as they can label sensitive small molecules and biomacromolecules under mild conditions without requiring high temperatures, extreme pH, or cytotoxic metal catalysts. Beyond their use in test tube (*in vitro*) radiolabeling, these bioorthogonal ligation methods are also being explored for *in vivo* pre-targeting strategies. This approach aims to improve the specificity of imaging and cancer radiotherapy in animal models [57,58,59].

Moreover, click chemistry allows the chelator to be radiolabeled independently before attaching it to the binding molecule, rather than labeling the entire chelator-targeting molecule complex. This method is beneficial because the radiolabeling process could potentially disrupt the structural integrity of the targeting molecule. Unlike alternative techniques, this type of ^177^Lu chelation provides benefits such as speed, high efficiency, and precise attachment to different targeting molecules. These click reactions are exceptionally selective, ensuring ^177^Lu binds only to a designated site on the targeting molecule, thereby reducing the likelihood of nonspecific interactions [51].

The identification of the most suitable class of chelators for the binding of ^177^Lu(III) ions plays a crucial role in the efficacy of the produced radioligands. As demonstrated by the ionic model, selection possibilities are considerably constrained, with optimal candidates being chelators that combine strong ionic binding capacity with the ability to form a complete coordination shell around the metal ion. Monodentate ligands are suboptimal for practical applications due to the omnipresent competition from hydroxide ions (OH^−^) in aqueous solutions, which form the most stable complexes with metal ions. Additionally, monodentate ligands cannot exploit the chelating effect, a key advantage offered by polydentate ligands [47]. Consequently, polycarboxylate-based chelators, attached to either carbon or nitrogen backbones, are commonly used in the synthesis of ^177^Lu radiopharmaceuticals for their superior binding properties. These ligands are generally categorized as either acyclic orcyclic polyaminopolycarboxylate-type chelators with 8 or 9 donor atoms. Among these chelators, DOTA (1,4,7,10-tetraazacyclododecane-1,4,7,10-tetraacetic acid) and its derivatives are the most widely used chelating agents for ^177^Lu, providing complexation of significantly high kinetic inertness and thermodynamic stability [20]. Table 4 demonstrates the structures of the most widely used ^177^Lu chelators employed in the development of bifunctional radioligands.

In the design of metal-chelators for biological systems, kinetic inertness and thermodynamic stability are paramount. Although acyclic chelators are conventionally considered less inert and faster-binding than their macrocyclic counterparts, this distinction is overly simplistic. Experimental evidence, particularly from X-ray crystallography, shows that trivalent radiometals (e.g., Ga(III), Y(III), In(III), Lu(III)) are not encapsulated by the macrocyclic cavity. The macrocycle’s key role is instead to support the carboxylic acid pendant arms, optimizing their interaction with the metal ion. Since this coordination geometry is consistent across these ions, the traditional classification of chelators into rigid cyclic and flexible acyclic categories may have limited fundamental relevance [47,60,61].

To clarify this point, the negatively charged oxygen atoms in polycarboxylate ligands create a powerful ionic attraction with the positively charged metal ion. This bond must be strong enough to displace the hydroxide (OH^−^) groups from the metal’s hydration sphere. This displacement is a key enthalpic driver that lowers the reaction’s free energy, making the process thermodynamically favorable. The ligand’s backbone—whether cyclic or acyclic—functions to pre-organize these carboxylate groups, positioning them near the metal ion to facilitate binding. While this pre-organization reduces entropy, the effect is counterbalanced by the release of water molecules from the metal’s hydration shell. Furthermore, the strong electrostatic attraction between the Lu(III) ion and the oxygen atoms provides a significant enthalpic gain. The net result is a substantial decrease in free energy, which is empirically demonstrated by the high stability constants of Lu(III) complexes with both open-chain and macrocyclic ligands (Table 5) [47,60,61].

The kinetic profile of the coordination reaction is directly influenced by the ligand’s structural rigidity. To achieve optimal metal binding, the ligand backbone must reconfigure, a process that is inherently more facile for flexible acyclic chelators than for conformationally constrained macrocyclic ones. This fundamental difference explains why macrocycles like DOTA require elevated temperatures for radiolabeling [47,60,61].

**Figure 4:**
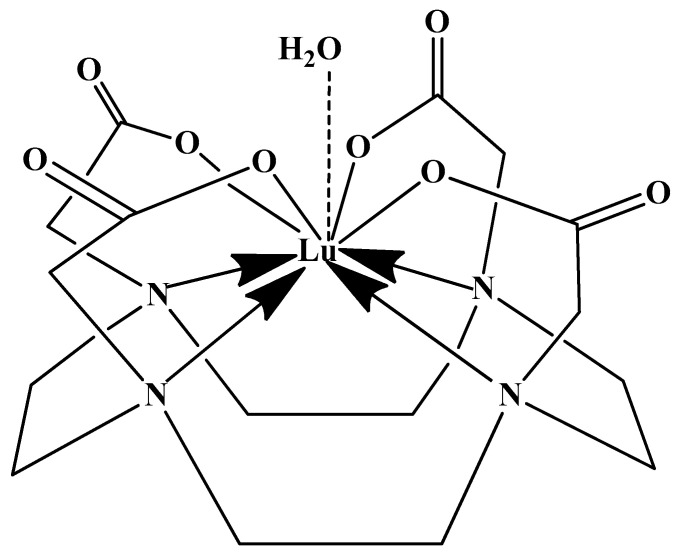
Schematic of the Lu(III) coordination sphere in the DOTA complex Na[Lu(DOTA)(H_2_O)]·4H_2_O, based on X-ray crystallographic data [60].

This thermal requirement is often misinterpreted as necessary for the metal to enter the macrocyclic cavity, suggesting a need for size compatibility. However, empirical evidence from X-ray diffraction reveals that the Lu(III) ion in the DOTA complex is not encapsulated within the ring but is capped by it (Figure 4). The actual function of the heat is to provide the energy needed to distort the macrocyclic ring into its active binding conformation. This distortion facilitates the proper spatial arrangement of donor atoms, including the ring nitrogens, to form the stable, square antiprismatic coordination sphere observed in the final complex [47,60,61].

In synthesizing bifunctional radioligands, standard approaches often functionalize a carboxylate group on DOTA for bioconjugation, which reduces its denticity and can destabilize the resulting metal complex. To circumvent this, platforms such as p-SCN-Bn-DOTA enable linkage via a non-coordinating side chain, thereby preserving the complete octadentate coordination sphere and the superior stability of the parent chelator [47,60,61].

Emerging research focuses on novel DOTA ligands where phosphinic acid groups substitute the traditional carboxylates, presenting distinct coordination behavior. Nevertheless, despite its slow complexation kinetics, DOTA continues to be the leading chelator for ^177^Lu radiopharmaceuticals. The pursuit of a derivative that enables rapid labeling under mild conditions remains an ongoing and unresolved challenge in the field [47,60,61].

The macrocyclic chelator NOTA is a hexadentate ligand that coordinates metal ions through its three ring nitrogen atoms and three pendant carboxylate oxygen atoms (N_3_O_3_-type). Compared to the larger DOTA macrocycle, NOTA’s smaller ring size requires less energy to distort into its optimal binding conformation, resulting in significantly faster radiolabeling kinetics. However, this comes with a trade-off: the N_3_O_3_ system provides only six donor atoms, which is insufficient to fully saturate the coordination sphere of the Lu(III) ion. This incomplete coordination can compromise the complex’s stability, a problem that is exacerbated when one carboxylate arm is used for bioconjugation, reducing the available donor atoms to just five. To resolve this limitation, the NETA chelator was developed. NETA incorporates an additional carboxylate group on a side chain, increasing its denticity. This design combines the fast labeling kinetics of an acyclic chelator with the enhanced *in vivo* stability provided by a macrocyclic framework [47,60,61]. It has to be noted that the octadentate NETA-trastuzumab conjugate instantly bound ^90^Y and ^177^Lu, forming radiolabeled complexes that remained stable in human serum and in tumor-bearing mice [62].

As a first-generation acyclic chelator, DTPA binds rapidly with various radiometals at ambient temperature. Its structure, consisting of a diethylenetriamine core with five carboxylate groups, allows it to act as an octadentate (N_3_O_5_) ligand. Despite a large body of stability data for DTPA complexes with isotopes from ^99m^Tc to lanthanides, the results are often difficult to generalize. Consequently, the efficacy of a novel DTPA-derived bifunctional radioligand must be validated on a case-by-case basis. This is illustrated by the unexpected stability difference between DTPA complexes of Y(III) and Lu(III). Despite their nearly identical coordination numbers and ionic radii, the Y(III) complex demonstrates superior stability, implicating other, less-easily measured physico-chemical parameters in the final complex stability [47,60,61].

Despite these limitations, researchers have developed novel DTPA derivatives to overcome its shortcomings. One such compound is 1B4M-DTPA, a bifunctional version featuring a methyl group on one of its carbon backbones. It is proposed that this methyl group introduces greater structural rigidity to the ligand, thereby enhancing the stability of its metal complexes [47,60,61]. An indicative example is the radiopharmaceutical Zevalin^®^, which is currently commercially available for radioimmunotherapy and was developed using 1B4M-DTPA. This analogue was selected for its efficient binding kinetics with the radioactive isotope ^90^Y [63].

In the design of radiotherapeutic agents for bone pain palliation, multidentate polyaminophosphonic acids serve as excellent carrier ligands. The therapeutic potential of ^177^Lu complexes with these radioligands was first explored in bone palliation agents with ^177^Lu-EDTMP [64], a finding later advanced into patient applications [65]. Their efficacy is attributed to their high affinity for skeletal tissue, selective localization to sites of heightened metabolic activity, and the formation of exceptionally stable *in vivo* chelates, particularly with radiolanthanides. Systematic evaluation of various cyclic and acyclic polyaminopolyphosphonate ligands complexed with ^177^Lu has revealed distinct advantages for cyclic structures. These cyclic phosphonates form complexes more efficiently, requiring lower ligand-to-metal ratios, and result in products with superior thermodynamic stability and kinetic inertness. Among acyclic ligands, EDTMP has shown effective complexation at a 20:1 ratio. A direct comparison between the ^177^Lu complexes of EDTMP and the cyclic DOTMP found that both could be synthesized in near-quantitative yields using low ligand concentrations. Biodistribution studies in Wistar rats indicated that both complexes have favorable pharmacokinetics. However, ^177^Lu-DOTMP cleared from the blood more rapidly and was retained less in the liver and kidneys than ^177^Lu-EDTMP. Conversely, ^177^Lu-EDTMP demonstrated consistently higher bone accumulation over time [66,67,68].

Nevertheless, the broader significance of ^177^Lu complexes with bifunctional radioligands was truly established with the development of ^177^Lu-DOTATATE, a radiopharmaceutical that targets somatostatin receptors for the treatment of neuroendocrine tumors [69,70]. Its success under the brand name Lutathera^®^ established PRRT as a major pillar in precision oncology.

### 2.2. ^177^Lutetium Production

**Figure 5:**
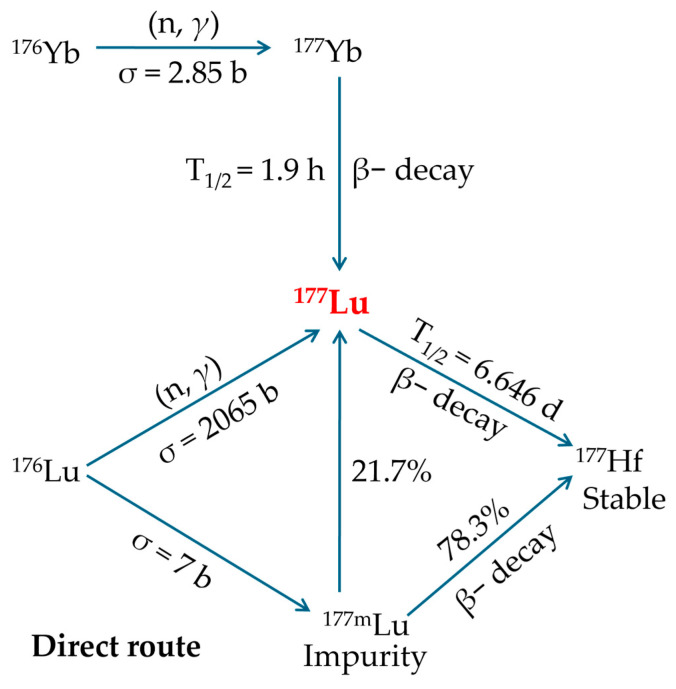
Dual distinct reactor-based production pathways for ^177^Lu generation. Reproduced with permission from Banerjee, S.; Pillai, M.R.; Knapp, F.F., Chem. Rev.; published by ACS, 2015 [19].

The growing availability and supporting data for ^177^Lu-based radionuclide therapy justify an evaluation of both existing and emerging production methods. While ^177^Lu can be generated using a cyclotron via charged particle acceleration, the most feasible and economical approach is through neutron irradiation in a nuclear reactor. The cyclotron-based method yields significantly lower radioactivity levels and is more costly, rendering it impractical for large-scale production. In a reactor, ^177^Lu can be produced through two primary pathways: direct neutron activation of enriched Lutetium-176 (^176^Lu) or indirectly by irradiating Ytterbium-176 (^176^Yb), which subsequently undergoes β− decay to form ^177^Lu (Figure 5) [19,44,71,72].

The direct, carrier-added production involves irradiating a stable ^176^Lu target (^176^L_2_O_3_) with neutrons according to the following nuclear reaction:


Lu176(n,γ)Lu177

To maximize yield and minimize the formation of undesirable radionuclides, the target is enriched in ^176^Lu [19,44,71,72].

This method offers the following benefits [19,44,71,72]:

- This is the most straightforward production method, requiring only minor adjustments to existing reactor and processing facilities.
- It employs a ^176^Lu_2_O_3_ target, which maintains excellent chemical and thermal stability during reactor irradiation.
- This route represents the most inexpensive option for obtaining ^177^Lu with the required purity.
- The nuclear reaction has a high cross-section (~2065 barn) and a neutron capture resonance integral of ~1087 barn, resulting in enhanced production yields and elevated specific activity, meaning just 1 mg of enriched ^176^Lu can yield roughly 50 patient doses.
- Significant specific activities of ^177^Lu are attainable by irradiating highly enriched targets in research reactors with flux levels in the medium-high range.
- Enables production scaling by simply modifying target dimensions to meet fluctuating demand.
- Post-irradiation processing of the target is straightforward, requiring only the conversion to ^177^LuCl_3_ and without the need for further refinement or preparation prior to clinical administration.

However, this approach has significant limitations [19,44,71,72]:

- Only about 30% of the irradiated ^176^Lu becomes the desired ^177^Lu, and this proportion decreases further due to radioactive decay before administration. Therefore, the highest achievable specific activity requires irradiation in high-flux reactor facilities.
- To significantly increase both the production yield and specific activity of ^177^Lu, it is essential to use targets enriched in the ^176^Lu isotope, as its natural abundance is only 2.6%.
- In practice, the specific activity of ^177^Lu from this route is 740–1110 GBq·mg^−1^, only about 18–27% of the theoretical 4.07 TBq/mg. This is because the product mixture contains just 25% radioactive ^177^Lu atoms, with the rest being stable lutetium isotopes. Therefore, the maximum achievable specific activity, even under ideal conditions in high-flux reactors, caps at around 70% of the theoretical limit.
- Directly produced ^177^Lu achieves an initial specific activity of 740–1110 GBq·mg^−1^ (20–30 Ci·mg^−1^), which may be suitable for PPRT. However, the continual decrease in specific activity due to radioactive decay limits its shelf-life, making it less ideal for procedures requiring consistently high specific activity.
- The process also generates ^177m^Lu, an unwanted beta-emitting byproduct with a 160-day half-life, complicating waste handling and disposal.

The production of ^177^Lu for therapy relies on the natural isotope ^176^Lu, which has a low abundance of 2.6% and a very long half-life. The process uses Lu_2_O_3_ as a target material for its stability and solubility. To obtain a high-specific-activity product suitable for therapy, it is essential to use targets that are both highly enriched in ^176^Lu and exceptionally pure. This high purity is critical because impurities can absorb neutrons in the reactor flux, diminishing the final product’s specific radioactivity [44].

The concentration of the ^177m^Lu impurity depends on both the irradiation time and the cooling period post-irradiation. Although the activity levels of ^177m^Lu are low due to its long half-life and low formation cross-section, its presence can raise regulatory concerns in some countries and creates a long-term radioactive waste issue. Nevertheless, studies have confirmed that the resulting increase in radiation dose from ^177m^Lu is negligible at clinical dose levels, particularly for PRRT. Under optimized production conditions, the ^177m^Lu/^177^Lu ratio is typically maintained at a very low 0.01% to 0.02% at the end of bombardment [44].

The practical maximum specific activity (SA) achievable via the direct production route is approximately 70% of the theoretical value, a level only attainable in high-flux nuclear reactors found in a limited number of countries. Reported SA values can reach 1850–2405 GBq·mg^−1^ (50–65 Ci·mg^−1^) in such facilities. However, the use of ^176^Lu-enriched targets (60–80%) in more widely available medium-flux reactors can reliably yield ^177^Lu with SA values of 740–1110 GBq·mg^−1^ (20–30 Ci·mg^−1^), which is adequate for all established radionuclide therapy applications [44].

Conclusively, although the patient radiation dose from the ^177m^Lu impurity is considered negligible, its management presents a significant logistical challenge for hospitals. The safe handling and long-term disposal of ^177m^Lu-contaminated waste are difficult to manage within standard hospital storage protocols. Despite this drawback, the direct production route remains the preferred choice for many facilities due to its cost-effectiveness and reliable supply of ^177^Lu in sufficient quality and quantity. This established supply chain is a critical foundation for expanding the radiopharmaceutical applications of ^177^Lu [44].

The indirect, no-carrier-added production utilizes neutron irradiation of ^176^Yb as the starting material. To optimize yield and minimize unwanted radionuclide formation, such as Ytterbium-169 (^169^Yb) and Ytterbium-175 (^175^Yb), the ytterbium is typically enriched to >99% in ^176^Yb. During irradiation, short-lived Ytterbium-177 (^177^Yb) (t_1_/_2_ < 2 h) forms and subsequently decays to ^177^Lu [19,44,71,72].


Yb176(n,γ)Yb177→β − decayLu177

This approach offers the following advantages [19,44,71,72]:

- After radiochemical separation from ^177^Yb, the final product contains only pure ^177^Lu with exceptionally high specific activity.
- The production pathway avoids creation of the problematic ^177m^Lu byproduct.
- SA does not depend on neutron flux.
- The radiochemical performance is sufficient.
- This production method yields ^177^Lu with extended viability (approximately 14 days) due to minimal SA loss over time.

However, this method presents some important challenges [19,44,71,72]:

- The nuclear reaction has a low cross-section (2.85 barn), requiring approximately 1 g of enriched ^176^Yb to generate activity equivalent to that produced from just 1 mg of enriched ^176^Lu in the carrier-added route. Therefore, to achieve sufficient yields and ensure efficient use of the enriched target material, neutron irradiation of the ^176^Yb_2_O_3_ target must be performed in a high-flux nuclear reactor.
- Using enriched targets with low neutron-capture cross-sections proves economically inefficient, as much of the material remains unactivated and requires expensive recovery and rigorous radiochemical processing to ensure complete ^176^Yb removal, further increasing complexity and cost.
- Extracting ^177^Lu from ^177^Yb is highly complex due to its extremely low concentration (~1 ^177^Lu atom per 5000 ^177^Yb atoms), necessitating an exceptionally efficient combination of purification methods, such as column chromatography, solvent extraction, supported liquid membrane extraction, extraction chromatography, or even electrochemical separation, cementation, and electro-amalgamation processes.
- The maximum achievable yield (saturation yield) of ^177^Lu requires irradiation lasting approximately 5–6 half-lives, typically necessitating several weeks of continuous neutron exposure. As a result, several factors contribute to substantially higher production costs, such as the requirement of isotopically enriched target material, larger irradiation volumes, extended irradiation periods, and complex post-irradiation radiochemical separation processes.
- This production method is significantly more expensive than alternative routes for obtaining ^177^Lu of the required purity.

Despite existing challenges, the potential of no-carrier-added ^177^Lu for TRT is significant, driving active research at many institutions. The success of this indirect production method hinges on a critical step: the efficient chemical separation of pure ^177^Lu from the much larger mass of the irradiated Yb target. This separation is particularly difficult because Yb and Lu share nearly identical coordination chemistry with the chelating agents used in radiopharmaceuticals. Therefore, evaluating the use of highly enriched ^176^Yb (up to ~97%) is not just promising but essential. Since very little of the target material is consumed during irradiation, developing a method to recover and recycle the unused, enriched ytterbium is a critical factor for making this indirect production route economically viable [44].

### 2.3. Cost-Effectiveness and Distribution Efficiency of ^177^Lu

A significant factor in the global adoption of ^177^Lu for therapy is its reliable production in high quantities and sufficient specific activity from numerous medium- to high-flux reactors worldwide. By optimizing irradiation parameters and using enriched targets, reactors with a neutron flux above 1 × 10^14^ n·cm^−2^·s^−1^ can consistently produce ^177^Lu with a specific activity exceeding 740 GBq·mg^−1^. This favorably contrasts with ^131^I, a widely used radionuclide, whose production—whether by neutron activation or fission—cannot achieve an isotopic abundance greater than 20% [19].

The indirect method for producing no-carrier-added ^177^Lu achieves a high theoretical SA but is an expensive process. This high cost stems from several factors: the price of the enriched ^176^Yb target material, low production yields due to a small nuclear cross-section, and the complex, costly procedures required to process the radioactive target and recover the valuable ^176^Yb for reuse. Because a significant portion of the expensive target is not activated, the process is often economically inefficient. Consequently, many countries are exploring the direct production route for ^177^Lu. The indirect method is typically reserved for situations demanding the highest SA, such as certain targeted therapies, where its benefits outweigh the substantial production costs [19].

The 6.7-day half-life of ^177^Lu is a key advantage for its widespread use in radiopharmaceuticals. This duration is ideal for clinical applications, allowing sufficient time for the drug to target and treat diseased cells. Furthermore, it provides significant logistical benefits for global distribution. The slow decay rate means minimal radioactivity is lost during transport from the production facility to distant hospitals and clinics. This efficiency helps keep ^177^Lu cost-effective and has been a major factor in its rapid adoption worldwide. To meet this growing demand, multiple suppliers now produce both carrier-added and no-carrier-added forms of ^177^Lu for drug development [19].

The current supply and demand for ^177^Lu is not entirely clear. While the approved drug Lutathera^®^ accounts for a significant portion of current use—estimated at 10,000–15,000 doses annually—this likely represents a fraction of total production due to extensive clinical research. Forecasting future demand is challenging, but analysts predict a dramatic increase, potentially several times over current levels. This growth is primarily driven by the adoption of prostate-specific membrane antigen (PSMA)-targeted therapies for prostate cancer. With approximately 366,000 global annual deaths from this disease, the potential patient population is vast, requiring multiple treatment cycles per patient. This suggests a potential market for ^177^Lu-PSMA therapy that is at least an order of magnitude larger than that for Lutathera^®^, indicating a substantial future demand even before considering other emerging applications [71].

The global market for ^177^Lu is highly concentrated, with the top three producers—Advanced Accelerator Applications (a Novartis company), Eckert & Ziegler Strahlen, and SHINE Technologies—collectively holding approximately 98% of the market share. Geographically, North America is the largest market, accounting for 44% of the total, followed by Europe (33%) and Asia-Pacific (18%). In terms of product and application, the market is dominated by the non-carrier-added type, which holds a 99% share, and its use in nuclear therapy, which constitutes about 98% of all applications. The ^177^Lu market size, estimations, and forecasts are provided in Table 6, in terms of output/shipments (Doses) and revenue (US$ millions), considering 2025 as the base year, with history and forecast data for the period from 2025 to 2032 [73,74].

### 2.4. ^177^Lutetium Clinical Advancements

Radionuclide therapy using therapeutic radiopharmaceuticals offers considerable benefits compared to conventional treatments, as it enables precise targeting of cancerous or diseased tissues while minimizing damage to surrounding healthy cells. Researchers have recognized the potential of ^177^Lu and have begun exploring its applications, particularly in oncology [19]. The combined action of the targeting molecule and the particle-emitting radionuclide enhances cell destruction, improving treatment efficacy for cancer and other conditions. Despite ongoing research into radiolabeled compounds for therapeutic use, a key hurdle remains in translating findings from preclinical animal studies to human clinical trials. However, following the approval of Lutathera^®^ and Pluvicto^®^ radiopharmaceuticals, extensive research is underway to explore various methods of binding and delivering ^177^Lu to different tumor types using diverse labeling techniques and biological mechanisms [19]. Table 7 presents a detailed overview of the most recent ongoing, terminated, or completed clinical trials with valuable results on the investigation of ^177^Lu radiopharmaceuticals under varying treatment conditions.

## 3. Magnetic Nano-Formulations

### 3.1. Magnetic Nano-Formulations as Theranostic Agents

The unique properties of nanomaterials—characterized by at least one dimension below 100 nm—have positioned them as transformative tools in modern medicine. Their distinct optical, magnetic, and electronic properties, coupled with an exceptional surface-to-volume ratio and tunable surface chemistry, render them particularly valuable for medical applications [102]. These materials are broadly classified into organic variants (liposomes, micelles, polymers) and inorganic forms (gold NPs, carbon allotropes, metal oxides), each with distinct preparation methods and structural characteristics.

Inorganic NPs are typically produced through precipitation or reduction reactions, while organic NPs form via self-assembly of molecular building blocks—with the notable exception of covalently structured dendrimers [103]. This fundamental difference in structure leads to varying stability profiles, with organic NPs exhibiting dynamic behavior in biological environments compared to their inorganic counterparts.

**Figure 6:**
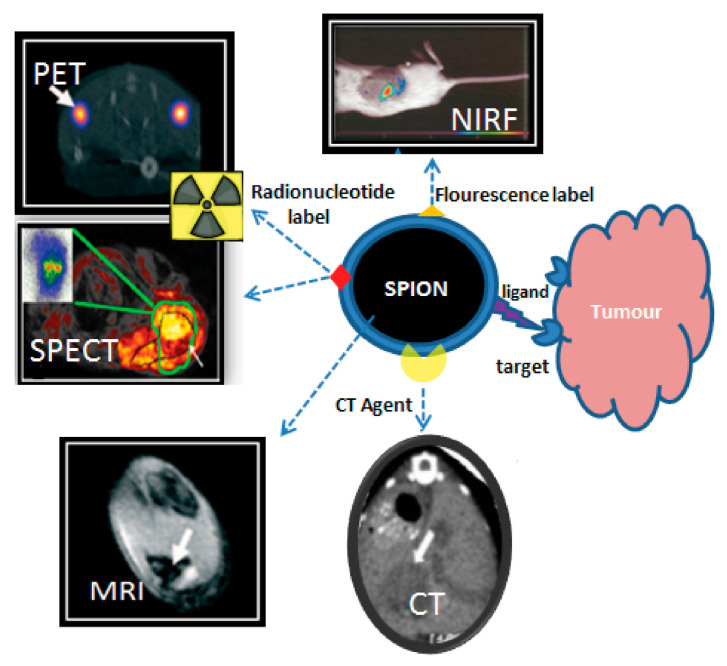
Theranostic applications of SPIONPs. Adopted from Thomas, R.; Park, I.-K.; Jeong, Y.Y. Int. J. Mol. Sci.; published by MDPI, 2013 [121].

The clinical success of liposomal and polymeric nano-formulations [104,105] has spurred interest in developing theranostic versions that combine therapeutic and diagnostic capabilities. However, the incorporation of imaging components (radioactive tracers, fluorescent dyes, MRI contrast agents) presents significant challenges, particularly regarding the potential dissociation of diagnostic and therapeutic components *in vivo* [106,107,108,109] (Figure 6). This has driven the search for intrinsically multifunctional nanomaterials that combine imaging capability, drug-loading capacity, and biological compatibility—especially important for cancer therapies requiring repeated administration [110].

Certain inorganic NPs demonstrate particular interest due to their inherent optoelectronic properties. These materials can serve dual roles as therapeutic enhancers [111] and diagnostic agents [112]. Among these types of NPs, iron oxide NPs (IONPs), including superparamagnetic IONPs (SPIONPs), magnetite (Fe_3_O_4_), and maghemite (*γ*-Fe_2_O_3_), are widely utilized for magnetic targeting of tumors or cells under an external magnetic field, facilitating drug delivery [113]. Their inherent magnetic properties, biocompatibility, and functional versatility render them valuable as MRI contrast agents for diagnostic imaging [114]. Additionally, IONPs show potential in multimodal therapies, combining radionuclide treatment, magnetic hyperthermia, and photothermal therapy [115]. A key advantage of IONPs is their versatile surface chemistry, which allows straightforward functionalization with biomolecules, including antibodies, peptides, nucleic acid structures, or other therapeutic agents to enhance targeting and efficacy, enabling precise interaction with overexpressed receptors on tumor cells and associated vasculature, representing a significant step toward personalized cancer theranostics [116].

Polymers and stabilizing agents as IONP coatings serve a dual function: they prolong blood circulation by inhibiting particle clumping and protein accumulation while also binding metal ions, eliminating the requirement for specialized chelating agents [117]. Furthermore, particle geometry optimization has enhanced circulation time and tissue penetration [118]. The development of ultra-small (<5 nm) IONPs has been particularly noteworthy, combining improved tumor accumulation via the permeability and retention (EPR) effect [119], with enhanced MRI contrast capabilities [120].

### 3.2. Radiolabeling of Magnetic Nano-Formulations

**Figure 7:**
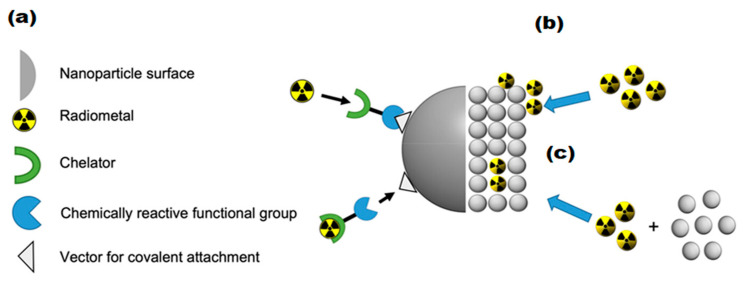
Visual representation of the three primary radiolabeling techniques for IONPs using positron-emitting isotopes: (**a**) Chelator-mediated approach—Utilizing bifunctional ligands to bridge NPs and radionuclides, (**b**) Surface adsorption method—Heat-driven deposition of radiometals onto NP surfaces, (**c**) “Hot + cold precursor” method—Simultaneous NP formation and radiolabeling through direct incorporation of radionuclides into the NP crystal lattice. Adopted from “Radiolabeled iron oxide nanomaterials for multimodal nuclear imaging and positive contrast magnetic resonance imaging (MRI): A review,” by Pellico, J.; Ruiz-Cabello, J.; Herranz F. *ACS Appl. Nano Mater.* **2023**, *6*, 20523–20538. CC-BY-NC-ND 4.0 [25].

Three principal methodologies exist for incorporating radionuclides into IONPs, with the chelator-based approach representing the most versatile and widely implemented technique. This method utilizes bifunctional ligands that serve as molecular bridges between the NP surface and metallic radionuclides, as illustrated in Figure 7a. Successful implementation of this strategy requires careful consideration of two fundamental aspects: the specificity of NP-ligand binding and the stability of radionuclide-chelate coordination [122,123].

The conjugation process must maintain the IONPs’ essential physico-chemical properties, including hydrodynamic size, surface charge, and magnetic characteristics. Conventional bioconjugation techniques typically employ amide bond formation between carboxyl and amine groups or thioether linkages through thiol-maleimide reactions [124]. While these methods offer the advantages of rapid conjugation under mild conditions with moderate to high yields, they often lack precise control over ligand density, which can inadvertently modify NP properties [125].

Recent advances in surface functionalization have introduced click chemistry approaches that provide superior control over conjugation processes [126,127]. These selective reactions, such as strain-promoted azide-alkyne cycloadditions and inverse electron-demand Diels-Alder reactions, enable more precise surface modifications. However, their application is limited by the requirement for specific complementary functional groups on both the NP surface and the ligand molecules [128,129].

A critical consideration in radiolabeling is the maintenance of radiochemical stability, which ensures the radionuclide remains coordinated to the NP under physiological conditions. Inadequate radiochemical stability can lead to radionuclide dissociation *in vivo*, potentially compromising imaging accuracy by generating signals from unbound radionuclides rather than the targeted NPs [130].

The field predominantly employs macrocyclic chelators, particularly DOTA and NOTA, due to their exceptional metal coordination properties. DOTA has gained widespread popularity for its ability to form stable complexes with various positron-emitting metals, though its requirement for high-temperature labeling conditions may pose challenges for biomolecule conjugation. NOTA, while operating effectively under milder conditions, exhibits a more restricted coordination sphere that limits its application to larger radionuclides such as ^132/135^La, ^131^Ba, ^201^Tl, ^203^Pb, ^213^Bi, ^223^Ra, and ^225^Ac [131].

These chelators are commercially available with diverse functional groups, facilitating their conjugation to NP surfaces. The chelator-based approach has demonstrated remarkable versatility, with numerous successful applications in T1-weighted IONP radiolabeling studies across various nano-formulations and imaging modalities. The continued refinement of these techniques promises to enhance the precision and reliability of radiolabeled IONPs for advanced diagnostic and theranostic applications [132].

In addition to chelator-based approaches, researchers have developed alternative methods for radiolabeling IONPs, including heat-induced radiolabeling (chemical adsorption) and the “hot + cold precursor” strategy. Heat-induced radiolabeling (Figure 7b) exploits the strong affinity of magnetite (Fe_3_O_4_) and maghemite (*γ*-Fe_2_O_3_) surfaces for certain radiometals, including ^89^Zr, ^69^Ge, and arsenic isotopes (^71^As, ^72^As, ^74^As). This radio-mineralization method involves heating IONPs in the presence of radiometals, leading to the deposition of radiolabeled metal oxides on the NP surface. While this technique achieves near-quantitative radiochemical stability, it typically requires high temperatures (>80 °C) [133].

On the other hand, the “hot + cold precursor” method (Figure 7c) enables simultaneous NP synthesis and radiolabeling in a single step. Unlike other techniques, this strategy requires careful optimization of reaction time due to its dependence on radionuclide half-life. Traditional IONP synthesis methods often take several hours, limiting compatibility with short-lived isotopes. The key advantage of the “hot + cold precursor” method lies in the direct incorporation of radionuclides into the NP crystal lattice, ensuring exceptional *in vivo* stability. Moreover, this technique minimizes structural modifications to the IONPs, preserving their original biodistribution profile, a critical factor for accurate imaging interpretation. When selecting a radiolabeling strategy, researchers must consider potential alterations to NP properties, as these could affect biological behavior and imaging results. In this regard, the “hot + cold precursor” approach offers distinct advantages by maintaining the native characteristics of IONPs while achieving robust radiolabeling [134].

## 4. ^177^Lu-Labeled Magnetic Nano-Formulations

### 4.1. Synthesis, Radio- and Physico-Chemical Characterization

The integration of ^177^Lu into magnetic nano-formulations presents unique physicochemical and radiochemical considerations that fundamentally influence their theranostic potential. While the nuclear properties of ^177^Lu are well-characterized for radiopharmaceutical applications, their manifestation in nanostructured systems introduces complex structure-property correlations that remain insufficiently explored. This section provides a comprehensive analysis of the critical parameters governing the performance of these hybrid systems, including: (i) the interplay between NP core composition and radiolabeling efficiency, (ii) the impact of surface functionalization on colloidal stability and physico-chemical characteristics, (iii) the retention of magnetic properties, and (iv) the evolution of these characteristics under physiological conditions.

In an effort to generate an effective theranostic agent to achieve optimal radiolabeling efficiency with a therapeutic and diagnostic isotope, Salvanou et al. [135] developed condensed colloidal nanocrystal clusters (co-CNCs) based on two variants of magnetic IONPs: alginate-coated nanocrystallites (MA) and their PEGylated counterparts (MAPEG), the latter being modified to confer stealth properties. The newly synthesized substrates of co-CNCs magnetic IONPs, prepared via a soft biomineralization process conducted at 50 °C under ambient pressure following the alkaline precipitation method of the starting ferrous precursor, were physico-chemically characterized in terms of their hydrodynamic diameter (D_h_) and surface charge (ζ-potential) via dynamic light scattering (DLS). The MA nano-formulations displayed an average D_h_ of 100 nm and a highly negative ζ-potential of −40 mV, consistent with the carboxylate-rich alginate coating. In contrast, the MAPEG NPs showed a slightly larger D_h_ of 120 nm and a near-neutral ζ-potential (−7 mV), reflecting the shielding effect of the PEG layer. The radiolabeling procedure for MA and MAPEG with ^177^Lu was performed through incubation of the nano-formulations with [^177^Lu]LuCl_3_ (50 µL, 10–30 MBq). Instant Thin Layer Chromatography-Silica Gel (ITLC-SG) analysis demonstrated high radiochemical yields of 95.21 ± 1.28% for [^177^Lu]Lu-MA and 93.65 ± 1.03% for [^177^Lu]Lu-MAPEG after 30 min of incubation. This efficient labeling likely results from the strong interaction between the Lu(III) cations and the negatively charged carboxylate groups (-COO^−^) in the alginate coating. The DLS measurements, which were performed in deionized H_2_O, revealed that [^177^Lu]Lu-MAPEG maintained its original D_h_ (≈119 nm) without any aggregation. Although the slightly higher pH conditions reduced immediate aggregation of [^177^Lu]Lu-MA (D_h_ ≈ 450 nm), these nano-formulations eventually precipitated after 24 h. The radiolabeled nano-formulations demonstrated excellent bench and *in vitro* stability when stored at room temperature, maintaining their integrity for at least 7 days post-preparation. Quantitative analysis performed via ITLC-SG, revealed retention rates of 93.97 ± 3.44% for [^177^Lu]Lu-MA and 95.60 ± 2.03% for [^177^Lu]Lu-MAPEG. In serum stability studies conducted at 37 °C (using a 1:10 v/v NP/serum ratio), both formulations showed gradual degradation over time. The [^177^Lu]Lu-MA sample decreased from 79.81 ± 1.28% intact at 2 h to 73.24 ± 2.59% after 7 days. Similarly, the [^177^Lu]Lu-MAPEG sample exhibited comparable stability, with intact percentages declining from 77.29 ± 1.69% (2 h) to 70.72 ± 1.75% (7 days post-labeling). Additional stability testing in aqueous solution (prepared at the same concentration used for biodistribution studies) showed remarkable stability, with >90% of the radiotracer remaining intact after 5 days of storage [135].

To overcome limitations of conventional brachytherapy, intratumoral administration of radionuclide-loaded nano-formulations (nanobrachytherapy, NBT) has emerged as a promising strategy for targeted radiotherapy of solid tumors, offering advantages over systemic intravenous delivery. To that end, Stanković et al. [136] developed ^177^Lu-labeled SPIONPs functionalized with meso-1,2-dimercaptosuccinic acid (DMSA) to create ^177^Lu-DMSA@SPIONPs as a potential nanobrachytherapy agent for localized tumor treatment. Following synthesis via the chemical co-precipitation of Fe(II)/Fe(III) cations, the structural properties of uncoated SPIONPs were analyzed using transmission electron microscopy (TEM). TEM images revealed a spherical nano-formulation morphology with an average diameter of 11.4 ± 3.2 nm and a polydispersity index (PDI) of 28.3% suggesting a moderate particle stability and a propensity for NP aggregation. X-ray diffraction (XRD) analysis of both uncoated SPIONPs and DMSA@SPIONPs demonstrated distinct crystalline patterns, representing a mixture of magnetite and maghemite phases. Crystallite size determination at 2θ ~36° yielded a mean value of 12.2 nm, showing excellent agreement with the TEM measurements. The D_h_ of both uncoated and DMSA-coated SPIONPs was evaluated using DLS. For uncoated SPIONPs, DLS analysis revealed a D_h_ of 46.1 ± 3.8 nm, which was notably larger than the core sizes obtained from TEM measurements (*p* < 0.05). This discrepancy likely resulted from NP aggregation in suspension, reflecting the system’s limited colloidal stability. Following DMSA coating, DLS measurements demonstrated a significant increase in D_h_ to 140.3 ± 6.5 nm (*p* < 0.05), consistent with the expected size expansion due to surface functionalization. The substantial difference between DLS and TEM-derived dimensions underscores the importance of considering both core size and hydrodynamic volume when characterizing NP systems. The ζ-potential measurements revealed that the uncoated SPIONPs exhibited a ζ-potential of +12.5 mV, which shifted to −35.1 mV after DMSA functionalization. This significant change to a negative potential reflects the introduction of carboxyl and thiol groups on the NP surface, substantially improving their suspension stability compared to bare SPIONPs. Fourier-transform infrared (FT-IR) spectroscopy provided further evidence of successful DMSA coating. The spectrum of DMSA@SPIONPs displayed characteristic absorption bands confirming the covalent attachment of DMSA molecules to the iron oxide surface. The spectral data support a proposed coating mechanism where (i) polar Fe-O-C bonds form through a condensation reaction with H_2_O elimination, and (ii) free thiol groups undergo oxidative coupling to form disulfide bridges in the coating layer. The DMSA-functionalized SPIONPs were effectively radiolabeled with ^177^Lu(III) ions using a direct labeling methodology. The radiolabeling procedure yielded 86.6 ± 2.1% incorporation efficiency, and subsequent magnetic separation produced ^177^Lu-DMSA@SPIONPs with radiochemical purity exceeding 99%, as verified by ITLC-SG analysis. The stability profile of the radiolabeled nano-formulations was systematically investigated under different storage conditions, including ambient temperature, human serum at 37 °C, and saline solution at 37 °C. Radiochromatographic monitoring over a 144 h period demonstrated excellent stability characteristics. The maximum observed release of free ^177^Lu(III) ions was limited to 2.2 ± 0.5% for room temperature storage, 3.6 ± 0.7% in human serum, and 4.2 ± 1.0% in saline solution at 37 °C [136].

The study of Ognjanović et al. [137] focused on the development of radiolabeled IONPs with a well-defined nanoflower-like morphology and precisely controlled dimensions for possible diagnostic applications or combined cancer treatment involving hyperthermia and radionuclide therapy. The IONPs were synthesized following a solvothermal approach of the polyol method, which involved alkaline co-precipitation of Fe(II)/Fe(III) salts in a solvent mixture of N-methyldiethanolamine and diethylene glycol. The synthesized IONPs were surface-coated with citric acid (CA), poly(acrylic acid) (PAA) or PEG to prevent aggregation while enhancing biocompatibility and facilitating straightforward radiolabeling. The morphology and size distribution of the IONPs were analyzed using TEM. The images revealed NPs with particle shapes between spherical and rounded cubes and an average size of 13.5 (±1.2) nm, which tended to form small agglomerates. The agglomerate sizes followed a log-normal distribution, with a D_h_ of 24.8 (±4.4) nm and a PDI of 18%. A combination of selected-area electron diffraction (SAED) and powder XRD analyses confirmed that the synthesized NPs likely consist of a mixed phase of magnetite and maghemite. The magnetic properties of the IONPs were comprehensively evaluated using multiple techniques. Isothermal magnetization measurements were conducted at both cryogenic (5 K) and room temperature (300 K) conditions, with applied fields reaching up to 3988.5 kA·m^−1^. The saturation magnetization (M_s_) values were determined to be approximately 77 emu·g^−1^ at 5 K and 70.5 emu·g^−1^ at 300 K. The room temperature magnetization curve exhibited characteristic superparamagnetic behavior, as evidenced by the zero-coercivity hysteresis loop passing through the origin. In contrast, at 5 K the IONPs demonstrated ferromagnetic properties with a measurable coercivity of 231.3 kA·m^−1^. This temperature-dependent magnetic behavior confirmed the superparamagnetic nature of the synthesized IONPs at physiological temperatures, while maintaining significant magnetic responsiveness at cryogenic conditions. The radiolabeling of the coated IONPs was performed by applying indirect radiolabeling approaches under mild conditions, taking advantage of the radionuclides’ ability to form stable complexes with functional groups present on the NP coatings. The methodology demonstrated several key advantages, including a simplified radiolabeling procedure applicable to multiple radionuclides, compatibility with various surface coatings, and the potential for combined diagnostic and therapeutic applications while maintaining NP functionality. The radiolabeling efficiency of coated IONPs with ^177^Lu exceeded 98% when conducted at 25 °C for 30 min, with no significant improvement observed upon extending the reaction time to 60 min. This high labeling efficiency was attributed to electrostatic interactions between the positively charged radionuclides and negatively charged functional groups on the NP surfaces: carboxylate groups in PAA@IONPs and CA@IONPs, and hydroxyl groups in PEG@IONPs. The PEG coating serves dual functions—its hydrophilic nature ensures aqueous dispersibility of the IONPs, while its steric hindrance effect prevents NP aggregation through interparticle repulsion forces. The radiolabeled coated IONPs were subjected to stability testing in both saline and human serum environments. Samples were analyzed using ITLC at predetermined intervals (1, 2, and 24 h post-preparation). Results demonstrated exceptional stability across all coating types (CA, PAA, and PEG@IONPs), with ^177^Lu-labeled NPs maintaining > 95% radiochemical purity in physiological media even after prolonged incubation (96 h). The effective functionalization of IONPs with PAA was verified through ζ-potential measurements, which showed a significant shift in the isoelectric point toward more acidic pH values. Specifically, the surface charge decreased from +14 mV to −26 mV at physiological pH (7.5), confirming successful PAA coating [137].

**Figure 8:**
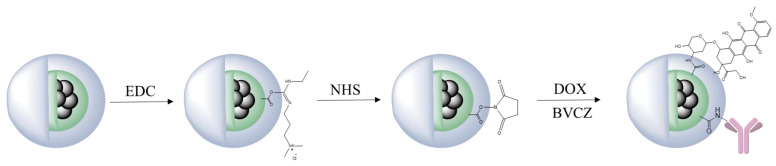
Functionalization scheme of PEG-stabilized AA-coated IONPs with DOX and BVCZ. Adopted from Salvanou, E.-A.; Kolokithas-Ntoukas, A.; Prokopiou, D.; Theodosiou, M.; Efthimiadou, E.; Koźmiński, P.; Xanthopoulos, S.; Avgoustakis, K.; Bouziotis, P. Molecules; published by MDPI, 2024 [138].

In an effort to support the notion that traditional cancer treatments like chemotherapy and radiotherapy, along with techniques such as brachytherapy, combined with the distinctive characteristics of IONPs, may lead to the creation of innovative theranostic agents, Salvanou et al. [138] assessed the capability of IONPs coated with alginic acid (AA) and PEG, functionalized with the chemotherapy drug doxorubicin (DOX) and the monoclonal antibody bevacizumab (BVCZ), to act as a nanoradiopharmaceutical for breast cancer treatment. The functionalization of the coated IONPs was achieved through the utilization of the carboxyl groups present in alginic acid. The coupling reaction was facilitated by the crosslinking agents N-ethyl-N′-(3-(dimethylamino)propyl)carbodiimide (EDC) and N-hydroxysuccinimide (NHS) at physiological pH (Figure 8).

The confirmation and quantification of the effective conjugation of the antibody and drug to the coated IONPs was performed via high-performance liquid chromatography (HPLC) and thermogravimetric analysis (TGA). The experimental results demonstrated high DOX loading efficiency in the nano-formulation, achieving 82.67 ± 3.87% by weight. Interestingly, PEG-stabilized AA-coated IONPs functionalized solely with DOX exhibited marginally greater encapsulation efficiency (88.99%) compared to those conjugated with both BVCZ and DOX (82.70%). Correspondingly, the conjugation efficiency of BVCZ to the alginate-coated NPs demonstrated substantial binding (41.84%) and encapsulation (35.97%) capabilities. The observed high loading capacities were attributed to the covalent bonding of the antibody and the drug to the coating of the IONPs. The TGA thermograms indicated a ~64.2% mass loss in the temperature range between 150 and 800 °C, which was assigned to the coating degradation in combination with the destruction of the conjugated DOX and BVCZ molecules. The particle size distribution and surface charge characteristics of the fabricated nano-formulations were analyzed through DLS measurements. The results revealed an average D_h_ of 474.6 ± 13.8 nm with a corresponding ζ-potential value of +16.7 mV. The measured high D_h_ and ζ-potential values were also attributed to the functionalization of the coated nano-formulations with the antibody and the drug. The *in vitro* DOX release under different physiological conditions was determined via reverse-phase high-performance liquid chromatography (RP-HPLC). Using phosphate-buffered saline (PBS) at both physiological (pH 7.4) and acidic (pH 5.5) conditions, initial drug release remained limited during the first hour (5.75 ± 0.93% and 6.69 ± 4.00%, respectively) mainly due to the conjugation of DOX, with only modest increases observed after 12 h (12.65 ± 0.42% and 12.06 ± 0.73%). Remarkably, the nano-formulations retained >70% of their drug payload (70.99% at pH 7.4; 71.51% at pH 5.5) following 72 h incubation at 37 °C. The introduction of pronase (a proteolytic enzyme targeting amide linkages between DOX and NPs) in acidic PB (pH 5.5) significantly altered the release kinetics. Drug liberation increased to 37.68 ± 3.94% by 12 h, demonstrating enzyme-mediated cleavage of drug-carrier bonds. Cumulative release progressed to 45.93 ± 6.32% at 24 h, 62.32 ± 7.38% at 48 h, and ultimately reached 90.94 ± 3.40% after 72 h. These findings clearly demonstrated pronase’s catalytic role in accelerating DOX release from the nano-formulation. The radiolabeling of the developed nano-formulations, functionalized and non-functionalized, occurred after direct incubation with ^177^Lu at 75 °C for 30 min. The radiolabeling yield and purity were determined via ITLC-SG. The measurements showed a 93.65 ± 1.03% yield for the ^177^Lu-labeled non-functionalized nano-formulations, which maintained 95.60 ± 2.03% stability after 7 days in serum (1:10 *v*/*v*, 37 °C). Correspondingly, the antibody-functionalized nano-formulations showed a radiochemical yield of 93.41 ± 2.59% after incubation at 40 °C and exhibited excellent *in vitro* integrity at room temperature, with 89.12 ± 2.99% remaining intact even after 14 days post-radiolabeling. In serum stability studies, conducted at physiological temperature (37 °C) using a 1:10 *v*/*v* NP-to-serum ratio, the conjugate demonstrated good stability over time with 84.80 ± 3.75% of the radiolabeled nano-formulations remaining intact after 1 day, gradually decreasing to 72.16 ± 3.05% by day 14 [138].

Gholami et al. [139] employed a chelate-free, heat-induced radiolabeling (HIR) method to develop ^177^Lu-labeled Feraheme (FH) nano-formulations for diagnostic imaging and radiotherapeutic applications. FH, an approved drug against iron anemia, consists of an iron oxide core of ~5 nm diameter coated with a thick carboxymethyldextran (CMD) layer, thus forming a nanoconstruct of ~25 nm overall diameter. The D_h_ of the synthesized FH NPs with incorporated non-radioactive Lu(III) was estimated via DLS at around 26.70 ± 0.45 nm, whilst the Lu(III) incorporation percentage exceeded 75% according to inductively coupled plasma mass spectrometry (ICP-MS) measurements. Taking into consideration that therapeutic applications have significantly different specific radioactivity requirements compared to imaging, the authors optimized radiolabeling by applying modifications to the HIR method, such as heated vortex mixing, temperature increase, short radiolabeling duration, or an increase in the initial radioactivity. In the case of ^177^Lu, the radiolabeling process was performed at a stable temperature (140 °C) under vortexing, the reaction duration was 2 h, and the initial radioactivity was around 15 MBq. Size exclusion chromatography (SEC) and TLC measurements demonstrated that ^177^Lu(III) cations reacted thermally with FH and resulted in high radiolabeling yield (91.0 ± 0.7%) and radiochemical purity (95.0 ± 0.7%) [139].

In an effort to showcase the broad applicability of Ligand Anchoring Group-Mediated Radiolabeling (LAGMERAL), a straightforward yet highly effective technique recently introduced for creating nuclear imaging nanoprobes, Ge et al. [140] developed ^177^Lu-labeled diphosphonate-polyethylene glycol (DP-PEG) coated Fe_3_O_4_ NPs suitable for MRI and therapeutic SPECT modalities. The Fe_3_O_4_ NPs, averaging 3.0 nm in size, were synthesized using a flow-based method involving the thermal decomposition of ferric acetylacetonate. Subsequently, the original hydrophobic oleate ligand was replaced with a diphosphonate-terminated PEG ligand (DP-PEG, M_n_ ≈ 2000), which features a methoxy group at the opposite end. Due to the strong binding affinity of diphosphonate groups for ferric ions, the resulting PEG-coated nano-formulations demonstrated excellent H_2_O dispersibility with no significant aggregation. DLS analysis revealed a narrow hydrodynamic size distribution centered at around 15.7 nm and a ζ-potential of 6.7 mV. MRI characterization showed that the nano-formulations exhibited longitudinal (r_1_) and transverse (r_2_) relaxivities of 7.8 and 32.7 m·M^−1^·s^−1^, respectively, at 3 T. Furthermore, the relatively low r_2_/r_1_ ratio (4.2) combined with the high r_2_ value indicated that these nano-formulations could serve as an effective dual-mode T_1_/T_2_ contrast agent for MRI applications. Subsequent radiolabeling with ^177^Lu resulted in an average radiolabeling rate of 50.9% and the experiments showed that the radiolabeling efficiency depends significantly on the ratio of radioactive atoms to NPs. Furthermore, the nano-formulations maintained excellent colloidal stability throughout the labeling process and subsequent purification steps, as evidenced by consistent relaxometric properties, hydrodynamic size distributions, and ζ-potential measurements before and after treatment. The radiolabeling stability was assessed by measuring with a gamma counter the radiochemical purity of radioactive nano-formulations in fetal bovine serum (FBS) after ultrafiltration. Following 6 h of incubation, the nano-formulations demonstrated excellent stability, with radiochemical purity levels at around 99.5%. Long-term stability tests revealed that ^177^Lu-labeled NPs maintained consistent purity (>99%) for over 72 h [140].

Rasaneh et al. [141] developed a novel dual-modality radiopharmaceutical for breast cancer treatment that enables simultaneous monitoring through both SPECT and MRI. DLS analysis revealed a D_h_ of 41 ± 15 nm, while the core size averaged 9.0 ± 2.5 nm. The radiolabeling efficiency was determined to be 61 ± 2%. Stability studies demonstrated that 86 ± 5% of ^177^Lu-trastuzumab remained intact in phosphate buffer, while 80 ± 3% was stable in human serum over 7 days. Additionally, the trastuzumab-functionalized nano-formulation conjugate exhibited high stability in phosphate buffer for 8 days, with only a 4% increase in size and no detectable free trastuzumab in PBS [141].

**Figure 9:**
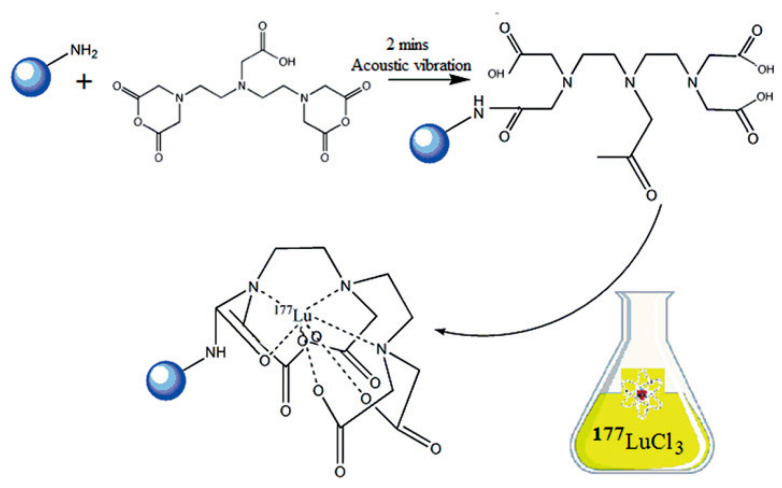
Conjugation of cDTPA to aminated dextran-coated IONPs and subsequent radiolabeling with ^177^Lu. Adopted with permission from Shanehsazzadeh, S.; Grüttner, C.; Yousefnia, H.; Lahooti, A.; Gholami, A.; Nosrati, S.; Zolghadri, S.; Anijdan, S.H.M.; Lotfabadi, A.; Varnamkhasti, B.S.; Daha, F.J.; Jalilian. A.R., Radiochim. Acta; published by De Gruyter Brill, 2016 [142].

Shanehsazzadeh et al. [142] assessed the suitability of SPIONPs radiolabeled with ^177^Lu as a promising theranostic agent for combined SPECT/MRI applications. The SPIONPs consisted of dextran-coated iron oxide cores crosslinked with surface-exposed amine (-NH_2_) groups. The bifunctional chelator cyclohexane-1,4-diyldinitrilo)tetraacetic acid dianhydride (ccDTPA) was coupled to the SPIONPs via a modified cyclic anhydride-mediated conjugation approach, using a 1:2 molar ratio (SPIONPs:ccDTPA) for optimal functionalization (Figure 9).

**Figure 10:**
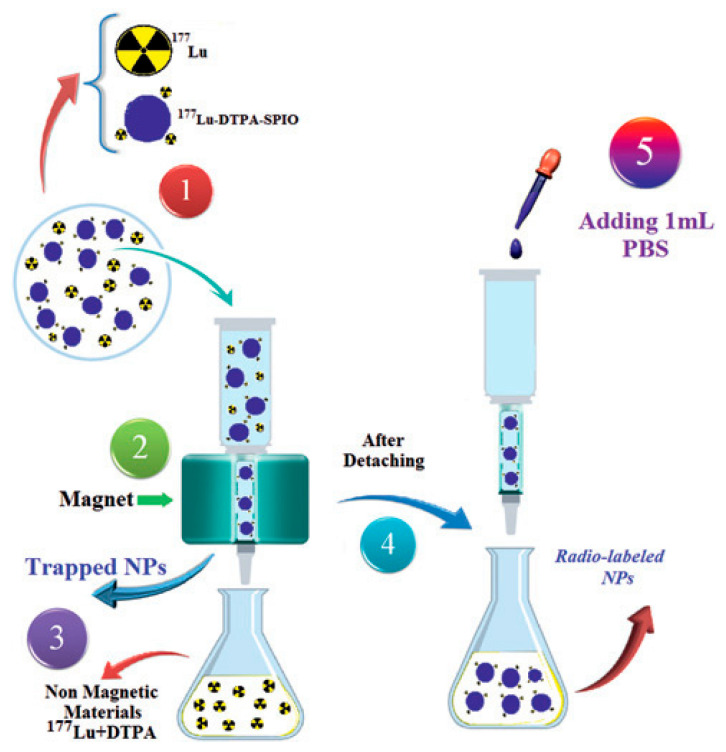
Descriptive scheme of the magnetic purification process of ^177^Lu-labeled functionalized nano-formulations via a MACS column to isolate the radiolabeled product from free ^177^Lu. ***Step 1***: The crude reaction mixture containing both ^177^Lu-labeled magnetic nano-formulations and free ^177^Lu is loaded onto the MACS column placed in a magnetic field. ***Step 2***: The column is washed with PBS to elute non-magnetic components, primarily free ^177^Lu. The MNPs are retained within the column. ***Step 3***: The flow-through containing the free radionuclide is collected and discarded. ***Step 4***: The column is removed from the magnetic field and allowed to stand for 1–2 min. ***Step 5***: Elution of the final product. Adopted with permission from Shanehsazzadeh, S.; Grüttner, C.; Yousefnia, H.; Lahooti, A.; Gholami, A.; Nosrati, S.; Zolghadri, S.; Anijdan, S.H.M.; Lotfabadi, A.; Varnamkhasti, B.S.; Daha, F.J.; Jalilian. A.R., Radiochim. Acta; published by De Gruyter Brill, 2016 [142].

After radiolabeling of the functionalized nano-formulations with ^177^Lu, a purification process was performed by employing a magnetic assorting cell separation (MACS) column to remove unbound ^177^Lu (Figure 10).

The functionalized nano-formulations exhibited a narrow size distribution with an average crystalline core diameter of ~9 nm according to TEM analysis. Photon correlation spectroscopy (PCS) analysis revealed a D^h^ of approximately 67 nm. Following radiolabeling, the magnetic nanoconstructs initially demonstrated 73% radionuclide purity, which increased to 98.5% after purification via MACS column separation. At 48 h post-labeling, the radiochemical purity remained high, with >93% stability in reference buffer and >78% in human plasma. D_h_ analysis confirmed that the nano-formulation size distribution remained unchanged after radiolabeling, indicating excellent colloidal stability [142].

To explore radiolabeling potential and design novel theranostic agents for combined cancer diagnosis and radionuclide-enhanced hyperthermia therapy, Mirković et al. [143] synthesized two types of MNPs: a poly-L-lysine-coated (PLL) magnetite system and an amino acid-decorated iron oxide platform. The amino acid-modified nano-formulations originated from perchloric acid-stabilized IONPs, which subsequently underwent surface engineering through adsorption of proline (Pro) and tryptophan (Trp) molecules. A systematic optimization study was conducted to determine the ideal Trp, Pro, and PLL loading on the MNPs. The loading was determined via Ultraviolet-Visible (UV-Vis) spectrometry. The measurements showed that the Pro formulation prepared with an input mass ratio of 5:1 demonstrated an adsorption efficiency of approximately 17% (*w*/*w*). In the case of Trp, the formulation prepared with a Trp-to-MNP mass ratio of 7:1 exhibited an adsorption efficiency of 3.5% (*w*/*w*). Correspondingly, the PLL formulation with a PLL-to-MNP mass ratio of 2:1 exhibited the highest PLL adsorption efficiency. A comprehensive suite of analytical techniques, such as TEM, DLS, differential centrifugal sedimentation (DCS), and thermomagnetic analysis, was employed to characterize the nano-formulations. TEM micrographs of the as-prepared samples revealed irregularly shaped particles with some degree of agglomeration attributable to sample preparation artifacts and average core diameters of 7–9 nm for non-functionalized, Pro- and Trp-functionalized formulations, respectively. The PLL-coated nano-formulations exhibited a slightly larger core diameter of approximately 11 nm. The DLS analysis showed that PLL MNPs displayed a monodisperse distribution with a peak at 112.6 nm, while uncoated, Pro-, and Trp-coated formulations showed respective peaks at 34.2 nm, 41.5 nm, and 49.7 nm. The observed size increases correlated well with DCS measurements (27.3 nm for uncoated, 53.8 nm for Pro-, 58.5 nm for Trp-coated MNPs) and confirmed successful amino acid functionalization. Notably, unmodified MNPs exhibited significant aggregation (D_h_ = 91.2 nm, PDI = 0.256), whereas PLL coating improved colloidal stability (PDI = 0.114), likely due to both the polymer’s molecular dimensions and its ability to form three-dimensional hydrophilic structures. Surface charge analysis via ζ-potential measurements revealed enhanced values for functionalized samples, confirming successful surface modification. All modified systems demonstrated excellent colloidal stability in 10 mM NaCl, with ζ-potential magnitudes exceeding 25 mV—the threshold for effective electrostatic stabilization. Magnetic characterization confirmed superparamagnetic behavior at 300 K for all samples, with no observable hysteresis. Saturation magnetization values were determined as 53.41 emu·g^−1^ (Pro MNPs), 47.11 emu·g^−1^ (Trp MNPs), and 57.17 emu·g^−1^ (PLL MNPs). The reduced magnetization relative to unmodified samples (uncoated MNPs = 60.6 emu·g^−1^, unstabilized MNPs = 75.6 emu·g^−1^) reflects the increased mass fraction of non-magnetic coating materials, as further supported by TGA. Magnetic core diameters derived from magnetization curves showed close agreement with TEM measurements, validating the consistency of the characterization methods. Under alternating magnetic fields (15.9 kA·m^−1^, 252 kHz), all functionalized nano-formulations generated heat within the therapeutic range (42–46 °C). PLL MNPs showed superior heating capacity with specific absorption rate (SAR) = 99.7 W·g^−1^ and intrinsic loss power (ILP) = 1.56 nH·m^2^·kg^−1^, significantly outperforming Pro MNPs and Trp MNPs. This enhanced performance stems from their optimized surface chemistry and magnetic properties, making PLL MNPs particularly suitable for magnetic hyperthermia applications. PLL MNPs demonstrated superior performance even under static magnetic field conditions. The radiolabeling of the produced nano-formulations with ^177^Lu (~50 μCi, 1.85 MBq) was performed at two different temperatures (room temperature and 80 °C) and pH 4.5–5, following a direct method. The exceptional colloidal stability of ^177^Lu-labeled formulations precluded radiochemical yield determination via magnetic separation. Consequently, radiochemical purity was assessed by ITLC-SG immediately post-labeling. Notably, PLL MNPs demonstrated superior radiochemical purity values (>98%) at both evaluated temperatures compared to the other functionalized formulations. Stability assessments of ^177^Lu-labeled PLL MNPs (80 °C radiolabeling) in physiological media demonstrated exceptional performance, maintaining >98% radiochemical purity in both saline and human serum at 37 °C over 96 h. Room temperature-labeled counterparts showed slightly reduced yet substantial stability, retaining > 80% integrity throughout the evaluation period [143].

Li et al. [144] engineered a novel NP system addressing key challenges in bladder cancer therapy through simultaneous mucosal penetration, reduced systemic exposure, and localized radiotherapy, offering potential for organ-preserving treatment strategies. More specifically, three types of hyaluronicacid(HA)-coated (HA molecular weights, 3K, 10K, and 90K Da) IONPs were synthesized via the coprecipitation method. The produced formulations were further colabeled with dibenzocyclooctyne (DBCO) and ^177^Lu. TEM images of the produced formulations revealed average diameters of 4.9, 6.5, and 7.0 nm, respectively. The transverse relaxivity (r_2_) of the nanoconstructs that was determined through MRI measurements confirmed the strong potential of the nanoprobes for T_2_-weighted MRI applications. The hydrodynamic sizes of the produced formulations were 21.2, 29.5, and 50.8 nm, respectively, indicating the effect of the HA’s molecular weight on the size of the formulated nanoprobes. Further increase in the D_h_ of the formulations was also observed after modification with DBCO. The organic content of the nanoprobes was determined via TGA analyses and the respective values for the three types of formulations were 67.1, 64.7, and 62.8%, respectively. Radiochemical purity was consistently >80% for all ^177^Lu-labeled nanomaterials, whilst stability monitoring in biological media (PBS and 10% FBS) via gamma counter demonstrated remarkable retention of radioactivity, with <10% loss observed throughout the evaluation period (72 h) [144].

### 4.2. Biological Applications

Recent advances in nuclear medicine and nanotechnology have converged in the development of radiolabeled nanoplatforms for targeted cancer imaging and therapy. For theranostic applications, radiolabeled inorganic NPs have emerged as promising agents for techniques like SPECT and MRI, providing critical data on pharmacokinetics and targeting efficiency. However, conventional cancer diagnostics face inherent limitations, including inadequate spatial resolution, insufficient soft-tissue contrast, and a lack of controlled drug delivery to specific sites. To address these challenges, SPIONPs functionalized with the theranostic radionuclide ^177^Lu have been developed. This combination enables multimodal, high-resolution imaging by enhancing MRI contrast while simultaneously permitting SPECT tracking, thereby facilitating integrated treatment planning and therapy [145].

**Figure 11:**
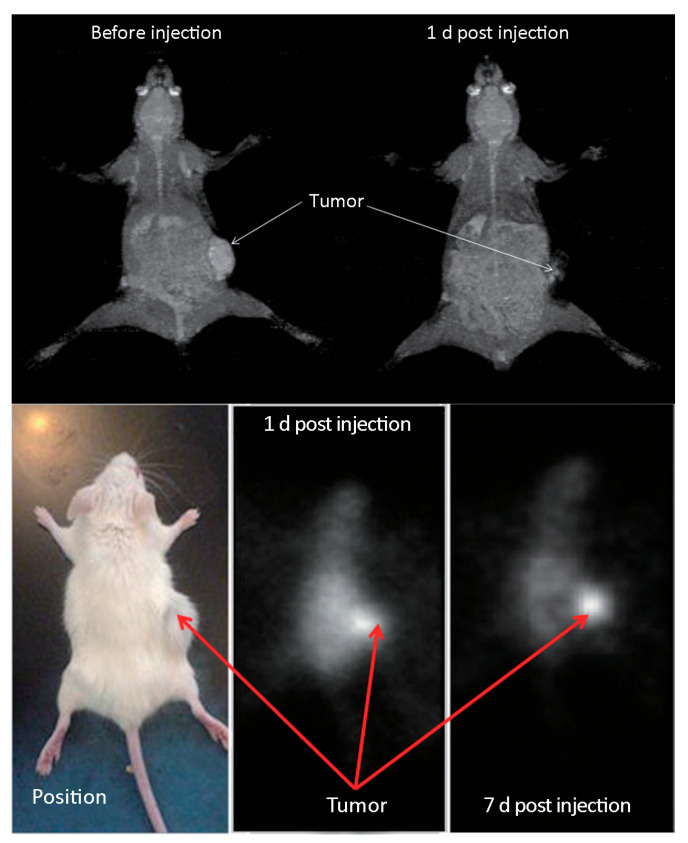
MRI images before and 1 day after injection of the ^177^Lu-trastuzuman-IONPs (top) and SPECT images at 1 and 7 days post injection of the ^177^Lu-trastuzuman-IONPs (bottom). The arrows indicate the tumors. Adopted from “Activity estimation in radioimmunotherapy using magnetic nanoparticles,” by Rasaneh, S.; Rajabi, H.; Daha, F.J. *Chin. J. Cancer Res.* **2015**, *27*, 203–208 [141].

An interesting *in vivo* approach in developing effective nanoprobes involved labeling of HER2-positive breast cancer inhibiting antibodies with ^177^Lu, followed by their attachment to IONPs to form contrast agents in MRI. Radiotherapy combined with imaging methods presents several limitations regarding the accuracy in organ volume measurements and radiopharmaceutical dose. In this context, the ^177^Lu-trastuzumab-IONPs developed by Rasaneh et al. [141] were evaluated as dual-activity agents for targeted radioimmunotherapy and effective monitoring of their biodistribution in mice with breast and liver tumors, utilizing different imaging methods. Despite the lower sensitivity of MRI relative to SPECT, ^177^Lu-trastuzumab-IONPs enabled enhanced MRI-based monitoring during radiotherapy. This facilitated superior dose estimation and significantly increased liver accumulation (~7% higher than the control NPs), while minimizing uptake in non-target organs (Figure 11). The combination of ^177^Lu’s therapeutic properties, trastuzumab’s targeted binding to overexpressed HER2 protein on cancer cells, and the imaging capability of IONPs opens new avenues for effective dosimetry tracking and tumor volume reduction.

SPIONPs with polymer surface coating represent an indicative example of enhanced contrast agents for MRI applications. It is well established that surface modification of MNPs offers enhanced biocompatibility, efficient delivery, and accumulation at specific target sites. To that end, the developed by Shanehsazzadeh et al. [142] promising DTPA-based ^177^Lu nanoplatform could be suggested as an effective theranostic agent for reticuloendothelial system (RES) studies employing SPECT and MRI. The size range and coating of the produced NPs were responsible for the accumulation in the liver and spleen and the rapid clearance from the blood. Compared with analogous SPIONPs targeting RES, accumulation of the ^177^Lu-labeled nano-formulations was increased from 56.64 ± 1.91 to 61.5 ± 2.9% in the liver and 12.5 ± 2.9 to 16.9 ± 1.4% in the spleen, respectively, probably due to their relatively small size, showing an increased absorption of ^177^Lu doses by RES compared to normal diagnostic nuclear medicine studies.

In another work, Hue et al. [146] developed ^177^Lu-labeled thermally cross-linked spherical SPIONPs (^177^Lu-TCL-SPIONPs) with enhanced stability (98%) and prolonged efficiency period (21 days) that were employed as diagnostic agents for *in vivo* cancer imaging. Upon labeling with ^177^Lu, TCL-SPIONPs were injected into ICR mice and distributed from systemic circulation into intracellular tissues. The ^177^Lu-labeled nano-formulations were mainly accumulated in the liver and spleen (36.121 ± 8.239% ID·g^−1^ at one day post-injection), in contrast to free ^177^Lu radioactivity that was accumulated in blood and various organs, mostly in the kidneys. ^177^Lu-TCL-SPIONPs radioactivity rapidly declined in blood, brain, and epididymis 28 days after injection. Interestingly, in relevant studies, a second peak of radioactivity was displayed by the spleen over a period of 60 days, indicating that the iron is slowly released and “restored” in the organ, hence handled as natural iron by the human body [147]. These findings were quite similar to the ones obtained using ^177^Lu-TCL-SPIONPs, confirming thus their safety as potential chemotherapeutic carriers.

**Figure 12:**
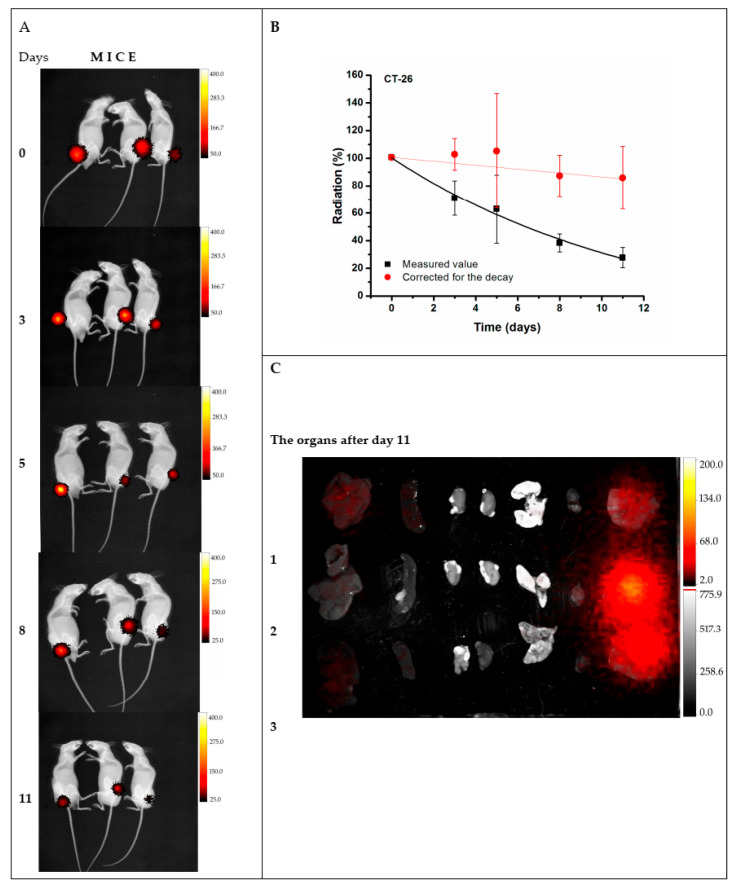
Biodistribution and pharmacokinetics of ^177^Lu-DMSA@SPIONPs following intratumoral injection in CT-26 tumor-bearing mice. (**A**) *In vivo* whole-body imaging (radioactivity-to-light) from day 0 to day 11 post-injection. (**B**) Pharmacokinetic profile of the nanomaterial within the tumor, derived from the integrated signal intensities in panel A. (**C**) *Ex vivo* quantification of radioactive signal in the tumor and major organs at day 11. Adopted from Stanković, D.; Radović, M.; Stanković, A.; Mirković, M.; Vukadinović, A.; Mijović, M.; Milanović, Z.; Ognjanović, M.; Janković, D.; Antić, B.; Vranješ-Đurić, S.; Savić, M.; Prijović, Ž. Synthesis, characterization, and therapeutic efficacy of ^177^Lu-DMSA@SPIONs in nanobrachytherapy of solid tumors. Pharmaceutics; published by MDPI, 2023 [136].

**Figure 13:**
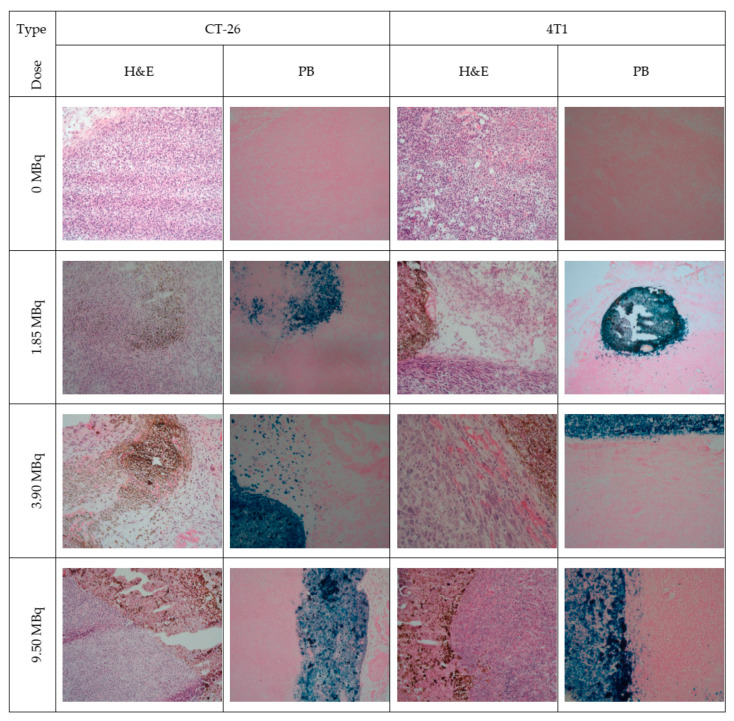
Photomicrographs of CT-26 and 4T1 tumor tissues following a single intratumoral injection of ^177^Lu-DMSA@SPIONs at doses of 1.85, 3.70, and 9.25 MBq. Tissue sections were stained with hematoxylin and eosin and Prussian blue. Scale bar represents 100 µm (200× magnification). Adopted from Stanković, D.; Radović, M.; Stanković, A.; Mirković, M.; Vukadinović, A.; Mijović, M.; Milanović, Z.; Ognjanović, M.; Janković, D.; Antić, B.; Vranješ-Đurić, S.; Savić, M.; Prijović, Ž. Synthesis, characterization, and therapeutic efficacy of ^177^Lu-DMSA@SPIONs in nanobrachytherapy of solid tumors. Pharmaceutics; published by MDPI, 2023 [136].

^177^Lu-labeled SPIONPs are considered indicative candidate nanoagents for nanobrachytherapy applications through direct injection in the tumor region. Compared to typical radiotherapy, the percentage delivered to targeted cancer cells is significantly higher, avoiding the rapid clearance by liver and spleen and the subsequent detrimental effect on these organs due to radioactivity accumulation. Stanković et al. [136] investigated the *in vivo* pharmacokinetic behavior of ^177^Lu-DMSA-SPIONPs after their intratumoral injection in colorectal CT-26 and breast 4T1 tumor-bearing mice. Evaluation of tumor growth following intratumoral administration revealed a significant, dose-independent suppression compared to controls. Examination of tumor tissue indicated moderate necrotic changes, most pronounced at the injection site. A key finding was the predominant localization and retention of the nanomaterial at the injection site in both tumor types without causing adverse side effects (Figure 12). *Ex vivo* biodistribution studies in CT-26 and 4T1 mouse tumor xenografts after variable radiation doses and number of treatments indicated high intra-tumoral radioactivity retention (90–95% ID for 1 day), and minimal leakage in both xenograft models for 14 days, with minimal radiation exposure to healthy organs and no observed general toxicity. Significant efficacy was achieved even at a low dose of 1.85 MBq, a finding with important implications for reducing radiation exposure to surrounding healthy tissues (Figure 13).

Magnetic nanomaterials open new avenues in the development of novel theranostics as contrast agents but also as carriers of drugs or radionuclides for radiotherapy and magnetic hyperthermia against cancer. Aiming to overcome limitations of radiotherapy and optimize NPs properties, such as prolonged blood circulation, aggregation, etc., several types of polymers have been utilized for surface modification.

A representative example can be found in the work of Salvanou et al. [135]. In their study, they performed a preliminary biological evaluation of two species of co-CNCs formulations based on: (i) surface-modified MIONPs with AA (MA), and (ii) stabilized MA NPs with PEG. *In vitro* evaluation of blood compatibility via hemolysis assay verified the safety profiles of both MA and MAPEG. Cytotoxicity assessment indicated that the unconjugated compounds were not markedly toxic to 4T1 cancer cells; however, the ^177^Lu-labeled MAPEG conjugate demonstrated a dose-dependent decrease in cell viability. Furthermore, the *in vivo* behavior of [^177^Lu]Lu-MAPEG revealed a high accumulation in the liver.

**Figure 14:**
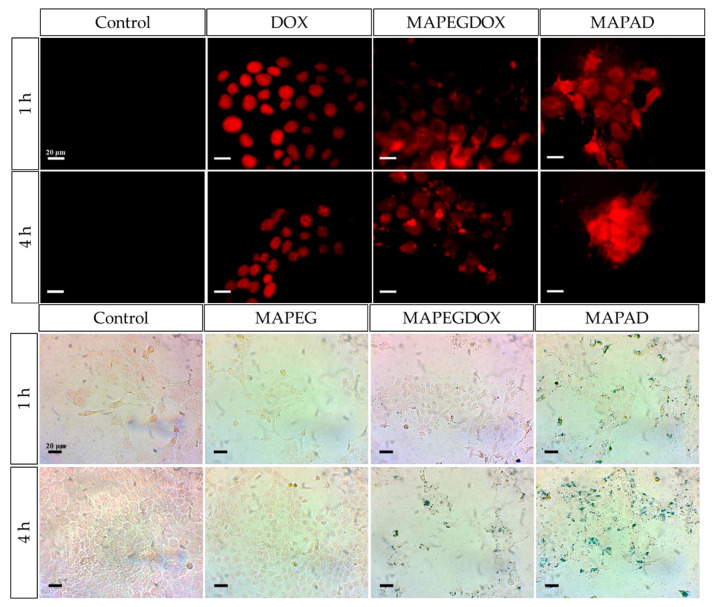
Cellular uptake in 4T1 cells after 1 h and 4 h of incubation with medium, free DOX, MAPEGDOX, or MAPAD, as shown by fluorescence microscopy (scale bar: 20 µm) (top) and Prussian blue staining of 4T1 cells revealed iron uptake after 1 and 4 h of treatment with medium, MAPEG, MAPEGDOX, and MAPAD (scale bar = 20 µm) (bottom). Adopted from Salvanou, E.-A.; Kolokithas-Ntoukas, A.; Prokopiou, D.; Theodosiou, M.; Efthimiadou, E.; Koźmiński, P.; Xanthopoulos, S.; Avgoustakis, K.; Bouziotis, P. Molecules; published by MDPI, 2024 [138].

These preliminary findings prompted the same research team to optimize MAPEG by functionalizing it with targeting ligands, aiming to develop a promising theranostic agent for locoregional delivery that enables substantial tumor accumulation. To that end, Salvanou et al. [138] examined a series of surface-modified species of MIONPs, such as: (i) MIONPs functionalized with the commonly used polysaccharide AA as chelator and the biocompatible polymer PEG (MAPEG), (ii) MAPEG with surface conjugated DOX and monoclonal antibody BVCZ (MAPAD), (iii) MAPEG with surface conjugated DOX (MAPEGDOX), as potential nanobracytherapy agents. The obtained nano-formulations were examined for their cytotoxicity against 4T1, MDA-MB-231, M165, MCF7, and SKBR3 cancer cells with varying levels of Vascular Endothelial Growth Factor (VEGF) expression. Evaluation via MTT assay indicated that the MAPEG NPs exhibited negligible toxicity up to 72 h post-treatment. The cytotoxic effects of both ^177^Lu-radiolabeled formulations, MAPEG and MAPAD, were found to be influenced by both the administered radioactivity and the duration of exposure, resulting in a time- and dose-dependent reduction in viability. Investigations into cellular uptake, using fluorescence microscopy and Prussian blue staining (Figure 14), revealed a marked difference between formulations. MAPAD NPs, functionalized with BVCZ, were internalized rapidly by 4T1 cells, with signs of nuclear localization observed after just one hour. Conversely, MAPEGDOX NPs showed predominantly cytoplasmic retention. *Ex vivo* biodistribution analysis up to 7 days post-injection compared three administration routes, with intratumoral delivery showing the most targeted and prolonged retention within the tumor. Ultimately, a single intratumoral administration of the functionalized ^177^Lu-labeled MAPAD formulations proved to have superior therapeutic efficacy in the 4T1 mouse model.

**Figure 15:**
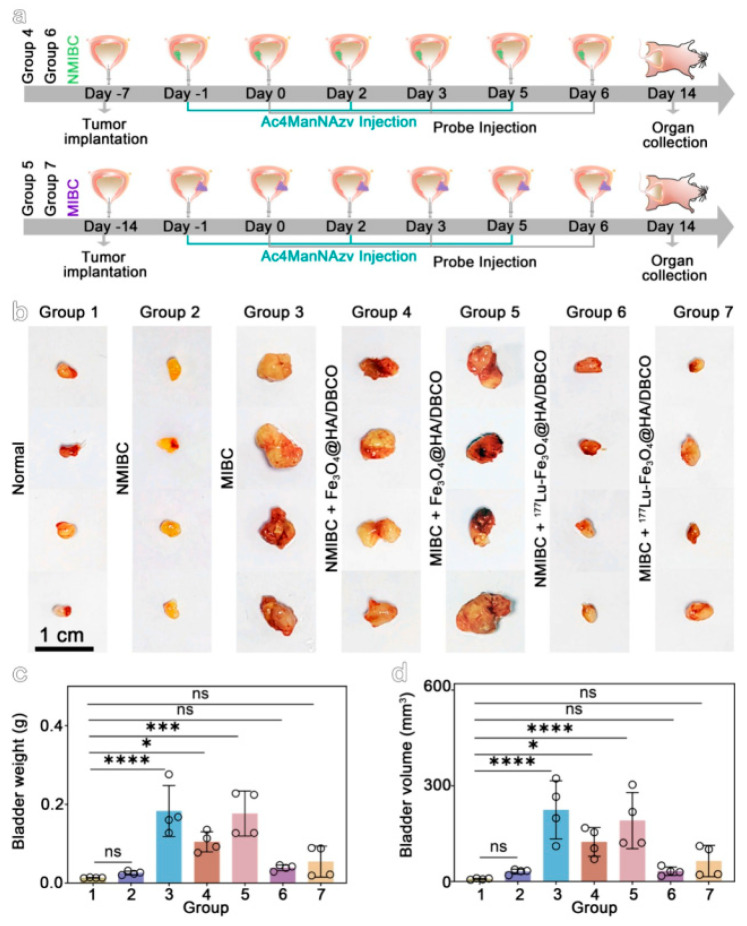
Evaluation of treatment efficacy in orthotopic bladder cancer models. (**a**) Schematic of the treatment schedules for NMIBC and MIBC models. (**b**) Macroscopic appearance of bladders isolated from the different experimental groups. (**c**,**d**) Bar graphs comparing bladder weight and volume. Data are presented as mean ± SEM. *p*-Values were calculated using one-way ANOVA with post hoc testing (* *p* < 0.05, ** *p* < 0.01, *** *p* < 0.001, **** *p* < 0.0001; ns, not significant). Adopted with permission from Li, Y.; Shan, S.; Zhang, R.; Sun, C.; Hu, X.; Fan, J.; Wang, Y.; Duan, R.; Gao, M. Imaging and downstaging bladder cancer with the ^177^Lu-labeled bioorthogonal nanoprobe. ACS Nano; published by American Chemical Society, 2024 [144].

**Figure 16:**
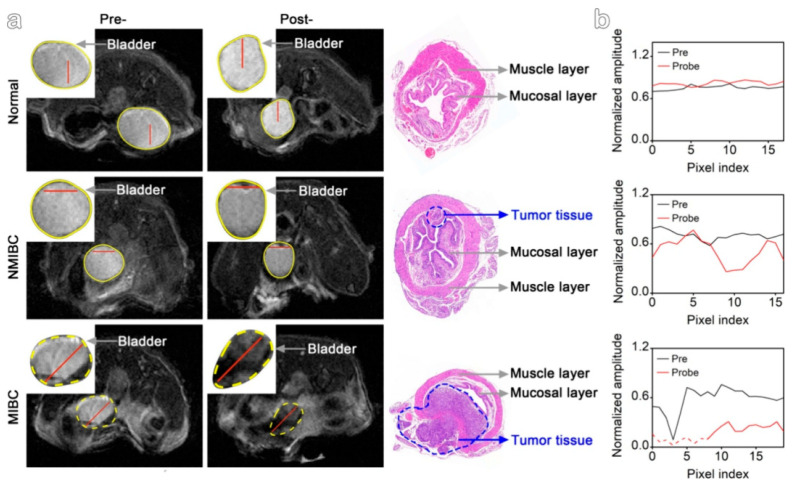
MRI and histological analysis of bladder tumors. (**a**) T2-weighted MR images acquired pre- and post-intravesical administration of the targeted contrast agent (N-Azidoacetylmannosamine-tetraacylated + Fe_3_O_4_@HA/DBCO). Corresponding Hematoxylin and Eosin-stained bladder sections confirm tumor invasion status, classifying tumors as NMIBC or MIBC. (**b**) Post-contrast MR image with the bladder highlighted (yellow circle). The red line indicates the location for line-scan analysis of MRI signal intensity (left panel). Adopted with permission from Li, Y.; Shan, S.; Zhang, R.; Sun, C.; Hu, X.; Fan, J.; Wang, Y.; Duan, R.; Gao, M. Imaging and downstaging bladder cancer with the ^177^Lu-labeled bioorthogonal nanoprobe. ACS Nano; published by American Chemical Society, 2024 [144].

Using a different polysaccharide, such as HA, Li et al. [144] employed ^177^Lu-Fe_3_O_4_@HA/DBCO nanoprobes with biorthogonality and mucoadhesivity for the effective diagnosis and treatment of bladder cancer. This therapeutic strategy combined the excellent biocompatibility and selectivity of HA and the capacity of DBCO to participate in *in vivo* biorthogonal reactions that overexpress artificial receptors on cancer cells, with the theranostic properties of ^177^Lu. *In vivo* studies carried out in mice with nonmuscle-invasive (NMIBC) and muscle-invasive bladder cancer (MIBC) suggested that ^177^Lu-Fe_3_O_4_@HA/DBCO nano-formulations could penetrate mucosa, reaching the region of the tumor, while Fe_3_O_4_@HA NPs could only adhere to mucosa. Likewise, Fe_3_O_4_@HA demonstrated lower inhibition of NMIBC and MIBC compared to the radiolabeled ^177^Lu-Fe_3_O_4_@HA/DBCO formulations, which caused significant tumor shrinkage, as illustrated in Figure 15. Additional biodistribution studies revealed the increased accumulation of ^177^Lu in bladders compared to other organs (Figure 16), indicating that this radiolabeled biorthogonal nanoagent improves the accurate detection of the tumor while increasing selectivity, specificity, and therapeutic efficacy.

In another study, the ^177^Lu-labeled surface-modified IONPs by Ognjanović et al. [137] were evaluated for their potential use in magnetic hyperthermia/radionuclide cancer therapy, and the results indicated that the ^177^Lu-PAA@IONPs exhibited notably low cytotoxicity at physiological levels. *In vitro* application of the NPs on CT-26 tumor cells, using magnetic hyperthermia, showed great performance (maximal effect at 116 kHz over a period of 30 min) with remarkably high ILP values (7.3 nHm^2^·kg^−1^), inducing a significant cytotoxic effect against cancer cells.

**Figure 17:**
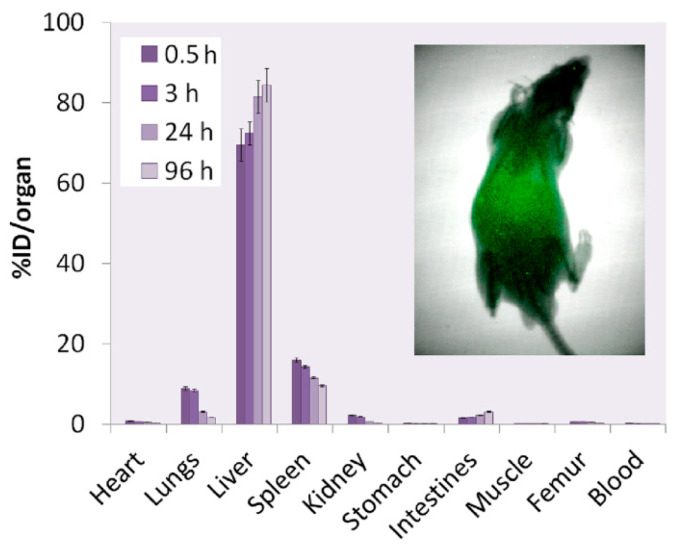
Biodistribution of ^177^Lu–PLL-MNPs in healthy Wistar rats at 0.5, 3, 24, and 96 h post-intravenous administration. Data represent the percentage of injected dose per organ (%ID/organ), expressed as the mean ± standard deviation (*n* = 3–5). The inset shows a radioimage of a rat 96 h after injection. Adopted with permission from Mirković, M.; Milanović, Z.; Perić, M.; Vranješ-Đurić, S.; Ognjanović, M.; Antić, B.; Kuraica, M.; Krstić, I.; Kubovcikova, M.; Antal, I.; Sobotova, R.; Zavisova, V.; Jurikova, A.; Fabian, M.; Konerack M. Design and preparation of proline, tryptophan and poly-L-lysine functionalized magnetic nanoparticles and their radiolabeling with ^131^I and ^177^Lu for potential theranostic use. Int. J. Pharm.; published by Elsevier, 2022 [143].

The observed high *in vitro* stability of the developed by Mirković et al. [143] ^177^Lu–PLL-MNPs urged the research team to further evaluate their *in vivo* behavior. The biodistribution of the ^177^Lu-labeled nano-formulations was evaluated in healthy Wistar rats at four time points (0.5, 3, 24, and 96 h) post-injection. The NPs accumulated primarily in the liver (69.45% ID at 0.5 h, increasing to 84.30% ID at 96 h) and the spleen (15.90% ID at 0.5 h, decreasing to 9.62% ID at 96 h), with minimal uptake in other organs (Figure 17). This pattern is typical for NPs of this size, which are rapidly cleared from the bloodstream by macrophages in the liver and spleen. The PLL coating enhanced the *in vivo* stability of the MNPs by preventing aggregation, as evidenced by low lung uptake. Furthermore, negligible radioactivity in the femur indicated that the ^177^Lu radiolabel remained stably bound to the PLL-MNPs, with no detectable free radionuclide release.

### 4.3. Analysis of Multimodal Functionality

The development of nanoscale theranostic agents that integrate multiple diagnostic and therapeutic functions into a single platform is a pivotal advancement in personalized medicine, particularly for oncology. In this context, the strategic importance of ^177^Lu-labeled MNFs lies in their inherent ability to exhibit synergistic multimodal activity for MRI, SPECT, and combined radiotherapy/hyperthermia. This multifunctionality allows for simultaneous and non-invasive anatomical localization via MRI, sensitive radionuclide-based tracking and dosimetry via SPECT, and the delivery of a powerful, localized therapeutic payload through the radioisotope’s beta-particle emissions complemented by magnetic hyperthermia. Such an all-in-one system enables real-time monitoring of biodistribution, precise tumor targeting, and the application of combinatorial treatments, thereby maximizing therapeutic efficacy while minimizing off-target effects and providing a comprehensive approach to cancer management.

Based on the first study of Salvanou et al. [135], it has been proven possible to create effective multimodal theranostic constructs by developing alginate and PEG-coated IONPs successfully radiolabeled with both ^68^Ga for PET imaging and ^177^Lu for therapy, thereby combining MRI capability from their superparamagnetic core with PET diagnostics and radionuclide therapy in a single agent. The incorporation of ^177^Lu provided several key features: it enabled efficient and stable direct radiolabeling via the alginate coating without a chelator, demonstrated significant dose-dependent cytotoxicity against cancer cells *in vitro*, and showed a favorable biodistribution profile *in vivo* with high and prolonged accumulation in RES organs like the liver and spleen, supporting its potential for therapeutic applications, particularly via locoregional administration.

Their second study [138] demonstrated the successful creation of a robust multimodal theranostic agent for MRI, SPECT, and therapy, based on an IONP core functionalized with DOX and BVCZ and labeled with ^177^Lu. From ^177^Lu, five critical features were leveraged: its beta emissions provided the primary therapeutic effect for tumor shrinkage, its gamma emissions enabled SPECT imaging and biodistribution tracking, its 6.7-day half-life allowed for prolonged tumor irradiation and logistical convenience, its stable direct radiolabeling to the NP’s alginate coating ensured a simple and efficient construction, and its application in a nanobrachytherapy approach via intratumoral injection resulted in exceptional tumor retention and minimized off-target effects, showcasing a potent and targeted combinational therapy.

Li et al. [144] demonstrated the successful creation of an effective multimodal theranostic agent for MRI, SPECT, and therapy, the ^177^Lu-Fe_3_O_4_@HA/DBCO nanoprobe. This system leverages the iron oxide core for T_2_-weighted MRI and the radioisotope ^177^Lu for both therapeutic and SPECT imaging capabilities. From ^177^Lu, five key features were harnessed: (i) its beta emissions enable potent targeted radionuclide therapy to shrink and downstage tumors, (ii) its gamma rays permit SPECT imaging and biodistribution tracking, (iii) its favorable half-life of 6.7 days allows for sustained diagnostic and therapeutic effects, (iv) its integration with a bioorthogonal targeting system ensures precise tumor accumulation, and (v) the treatment was shown to inhibit metastasis, highlighting its comprehensive therapeutic potential.

Stanković et al. [136] developed an effective multimodal theranostic agent for MRI, SPECT, and therapy using SPIONPs coated with DMSA and radiolabeled with ^177^Lu. The SPIONP core provides the inherent superparamagnetic properties necessary for MRI, while the^177^Lu radionuclide contributes several critical features: (i) its beta emissions deliver the primary therapeutic effect via a localized “cross-fire” within a ~2 mm range in tissue, (ii) its gamma emissions enable SPECT imaging and biodistribution tracking, (iii) its 6.7-day physical half-life allows for prolonged tumor irradiation and logistical convenience in handling, (iv) it achieves stable, direct chelator-free radiolabeling via the DMSA coating with high yield and *in vitro* stability, and (v) when administered via intratumoral injection (nanobrachytherapy), it results in exceptional tumor retention, minimal systemic leakage, and high therapeutic efficacy without signs of general toxicity, demonstrating a potent and targeted combinatorial approach.

Excellent multimodal theranostic systems for MRI, SPECT, and combined therapy using polyol-synthesized, flower-like IONPs coated with PAA were developed by Ognjanović et al. [137]. The SPIONP core provides the strong T2 contrast capability for MRI, while the successful radiolabeling with^99m^Tc enables SPECT imaging. For therapy, the system leverages multiple features from ^177^Lu: (i) its beta emissions enable radionuclide therapy, (ii) it achieves very high radiolabeling yields (>98%) via a simple, indirect method with the PAA coating, (iii) it exhibits excellent *in vitro* stability with over 95% retention after 96 h in physiological media, and (iv) it is used in conjunction with the NP’s outstanding magnetic hyperthermia capability (intrinsic loss power of 7.3 nH m^2^·kg^−1^), creating a potent dual hyperthermia/radionuclide therapeutic platform.

Gholami et al. [139] demonstrated the successful development of effective multimodal constructs for MRI, SPECT, and therapy using the chelate-free HIR method with FH NPs. This platform successfully incorporates a variety of diagnostic and therapeutic isotopes, including ^177^Lu, into the iron oxide core without the need for chelators, preserving the NP’s magnetic properties for MRI while enabling radiolabeling for nuclear imaging and targeted radionuclide therapy. For^177^Lu, the study demonstrated high radiochemical yields (RCY ≈ 91%) and purity (RCP ≈ 95%), confirming efficient and stable binding under HIR conditions. Additionally, the ^177^Lu-FH NPs exhibited minimal changes in size and relaxivity, maintained *in vivo* stability, and showed potential for combination with other modalities, supporting their use as versatile theranostic agents.

Based on the research presented by Ge et al. [140], effective multimodal nanoprobes for MRI, SPECT, and therapy using the universal LAGMERAL method were developed. This approach successfully integrated various functional inorganic NPs, such as Fe_3_O_4_ for MRI and Cu_2-x_S for photoacoustic imaging, with different radionuclides. For the therapeutic isotope ^177^Lu, the system demonstrated several key features: (i) it achieved a radiolabeling yield of 67.4% on Cu_2-x_S NPs, (ii) provided excellent radiolabeling stability with 99.5% purity retained in serum after 72 h, (iii) enabled high-contrast SPECT imaging for sentinel lymph node detection, and (iv) combined the radiotherapeutic effect of ^177^Lu with the intrinsic photothermal properties of the NP carrier for potential synergistic therapy.

The biodistribution study by Hue et al. [146] demonstrated that the ^177^Lu-TCL-SPIONPs show a clear potential for use in multimodal systems combining SPECT and therapy. The primary feature of ^177^Lu-TCL-SPIONPs is their distinct and stable biodistribution profile, characterized by high and sustained accumulation in the liver (36.12% ID·g^−1^ at day 1, 15.15% ID·g^−1^ at day 28) and spleen (15.62% ID·g^−1^ at 0.5 h, 9.09% ID·g^−1^ at day 28), which are organs of the RES. This pattern, coupled with rapid clearance from the blood and most other tissues, confirms the NP’s role as a carrier that can be tracked via the gamma emissions of ^177^Lu for SPECT imaging. Furthermore, the inherent superparamagnetic property of the iron oxide core provides the functionality for MRI, while the beta-particle emission from ^177^Lu delivers the therapeutic effect. Therefore, the system effectively integrates three key features from ^177^Lu: (i) its role as a SPECT radiotracer for imaging biodistribution, (ii) its function as a beta-emitter for radiotherapy, and (iii) its stable attachment to an MRI-active NP, creating a promising theranostic platform.

Rasaneh et al. [141] showed the successful development of a highly effective multimodal construct for MRI, SPECT, and therapy, the ^177^Lu-trastuzumab-IONPs platform. This system leverages three key features from^177^Lu: (i) its gamma emissions enable precise tracking of the NP’s biodistribution via SPECT imaging, (ii) its beta-particle emission provides the therapeutic mechanism for radioimmunotherapy, and (iii) its stable conjugation to the antibody-NP complex ensures targeted delivery. Crucially, the IONP core simultaneously serves as a potent T2 contrast agent for high-resolution MRI. The study demonstrated that this combined approach allowed for more accurate activity estimation and dosimetry in tumors compared to SPECT alone, confirming the construct’s role as a superior theranostic platform.

As demonstrated by Shanehsazzadeh et al. [142], the ^177^Lu-DTPA-SPIONPs system successfully harnesses three key features from^177^Lu: (i) its gamma emissions enable precise biodistribution tracking via SPECT imaging, (ii) its beta-particle emissions provide the therapeutic mechanism for targeted radiotherapy, and (iii) its stable conjugation via a DTPA chelator ensures the radionuclide remains attached to the NP carrier. Crucially, the SPIO core serves as a potent T2 contrast agent for high-resolution MRI. The construct exhibited a favorable biodistribution profile, with high and sustained accumulation in the liver and spleen and rapid clearance from the blood, confirming its potential as a promising theranostic platform for RES-targeted applications.

Mirković et al. [143] created effective multimodal nanoconstructs suitable for MRI, SPECT, and combination therapy. The PLL-MNPs exhibited superparamagnetic behavior essential for MRI, were successfully radiolabeled with the theranostic radionuclide ^177^Lu, and demonstrated a high SAR for magnetic hyperthermia therapy. The ^177^Lu labeling provided multiple key features: (i) it enabled SPECT imaging capabilities due to its gamma emissions, (ii) delivered beta-particle therapy for localized tumor treatment, (iii) allowed for diagnostic dosimetry, and (iv) exhibited excellent *in vitro* and *in vivo* stability, which is critical for ensuring the radionuclide remains attached to the nanocarrier during diagnostic and therapeutic procedures.

## 5. Current Challenges

^177^Lu-labeled magnetic nano-formulations (^177^Lu-MNFs) represent a compelling theoretical platform for theranostic oncology, combining the imaging and therapeutic potential of a radionuclide with the targeting and hyperthermia capabilities of MNPs. However, their clinical translation remains a distant goal, hindered by fundamental challenges that must be systematically addressed in future preclinical studies. The critical hurdles of inherent magnetic core limitations, dosimetry, scalability, biocompatibility, and regulatory approval currently define the essential research agenda for this field.

A fundamental set of constraints arises from the inherent properties of the magnetic cores. The efficacy of magnetic targeting and hyperthermia is highly dependent on core characteristics such as composition, size, crystallinity, and magnetic saturation. A significant limitation is the potential for oxidative degradation of certain cores (e.g., magnetite) in the physiological environment, which can compromise magnetic functionality and release potentially toxic ions. Furthermore, achieving sufficient magnetic responsiveness for effective *in vivo* targeting against the dynamic forces of blood flow remains a major physical challenge. Future research must focus on developing more stable, high-performance magnetic materials (e.g., doped ferrites) and rigorously evaluating their structural integrity and magnetic performance post-synthesis and under physiological conditions.

A significant and complex challenge is the accurate prediction of radiation dosimetry. The unique biodistribution of ^177^Lu-MNFs, characterized by high and persistent accumulation in organs of the mononuclear phagocyte system (e.g., liver, spleen), could lead to unintended radiation doses to these healthy tissues. Preclinical studies must prioritize developing sophisticated pharmacokinetic models that can reliably extrapolate organ absorption and radiation doses from animal models to humans, a critical step for ensuring patient safety in future trials.

A primary obstacle is the absence of robust, scalable manufacturing protocols. The synthesis of ^177^Lu-MNFs with precise control over the critical magnetic core properties, in addition to size and surface chemistry, is a laboratory-scale achievement. Reproducibly scaling this synthesis under Good Manufacturing Practice (GMP) conditions for clinical-grade material is a significant unsolved problem. Therefore, a paramount future direction is to develop continuous and controlled synthesis methods that guarantee batch-to-batch consistency, a prerequisite for generating reliable preclinical data and eventual regulatory approval.

Furthermore, the biocompatibility and *in vivo* pharmacokinetics of these complex agents are largely unknown. Key questions regarding their stability in the bloodstream, potential for off-target accumulation, and long-term clearance pathways remain unanswered. The chemical stability of the magnetic core is directly linked to these safety concerns. Before any clinical application can be considered, extensive preclinical investigations are mandatory. These must focus on engineering coatings that minimize immune recognition and on conducting comprehensive toxicological studies to establish a foundational safety profile, which is intrinsically linked to the dosimetry concerns.

Finally, the regulatory pathway for ^177^Lu-MNFs is exceptionally complex due to their hybrid nature as combination products. The lack of standardized characterization methods for evaluating magnetic properties, radiochemical purity, stability, and sterility specific to these formulations presents a major barrier. A critical future direction involves proactively collaborating with regulatory agencies to define the necessary criteria, including robust dosimetry data and magnetic performance metrics, and develop the analytical protocols required to evaluate these novel agents, thereby creating a clear roadmap for development.

## 6. Future Perspectives

The development of ^177^Lu-labeled MNFs represents a compelling frontier in targeted radionuclide therapy, merging the cytotoxic power of beta radiation with the unique capabilities of nanotechnology. These “theranostic” agents are engineered to be guided by external magnetic fields to specific tumor sites, while their intrinsic magnetic properties allow for non-invasive tracking via MRI. The central hypothesis is that this dual-targeting approach—passive accumulation through enhanced EPR and magnetically controlled active guidance—could dramatically improve tumor dose delivery while minimizing irradiation of healthy tissues. This promises a significant leap beyond current ^177^Lu-labeled molecules, potentially unlocking treatments for cancers that are currently inaccessible.

Looking beyond the translational challenges, future perspectives for ^177^Lu-MNFs are expansive and hinge on advanced nanoscale engineering. Next-generation platforms could be designed for combination therapies, such as the co-delivery of chemotherapeutic agents or immunomodulators to synergize with radiotherapy and magnetic hyperthermia, potentially overcoming treatment resistance. Furthermore, engineering MNFs for multimodal imaging, by incorporating contrast agents for MRI or fluorescent tags, could provide complementary diagnostic information, enhancing tumor localization and treatment planning.

Perhaps the most promising direction lies in developing smart, stimulus-responsive designs. These “intelligent” MNFs could be engineered to release their therapeutic payload or enhance radiation dose uptake specifically in response to the unique tumor microenvironment (e.g., low pH, specific enzymes) or an externally applied stimulus (e.g., alternating magnetic field), thereby maximizing efficacy while minimizing systemic toxicity revolutionizing thus the treatment of hard-to-reach or radioresistant solid tumors, such as certain brain, pancreatic, or advanced prostate cancers, where precise localization is critical.

A promising approach involves functionalizing ^177^Lu-labeled NPs with targeting ligands—such as peptides, antibodies, human serum albumin, or biomimetic coatings like exosomes. This functionalization enhances both the biocompatibility and tumor-specific targeting of the platform. For instance, using a protein scaffold for ^177^Lu-MNFs can increase tumor accumulation while minimizing off-target cytotoxic effects, thereby concentrating the therapeutic radiation within the tumor tissue.

However, the path from concept to clinic is fraught with formidable scientific and regulatory hurdles. Key challenges include achieving scalable and reproducible synthesis of nano-formulations with uniform size, stable radiolabeling, and consistent magnetic properties. The biological behavior, including long-term biodistribution, potential immune response, and eventual clearance of these inorganic particles from the body, must be thoroughly characterized to ensure safety. Furthermore, the practical efficacy of magnetic targeting in deep-seated human tumors remains to be conclusively demonstrated, and navigating the regulatory pathway for a novel combination product (drug + device) will be complex and costly.

Despite the challenges, the question is not whether to abandon this research, but how to strategically advance it. Researchers should not “forget about it”; instead, they should focus their efforts with precision. Priority should be given to interdisciplinary collaboration between radiochemists, materials scientists, and clinicians to tackle the core issues of manufacturing and safety. Research must move beyond proof-of-concept studies to robust, pre-clinical models that realistically test targeting efficacy and therapeutic superiority over standard care. The goal should be to generate compelling data that de-risks the technology for larger-scale investment and clinical translation.

In conclusion, the pursuit of ^177^Lu-labeled MNFs is not just a niche interest but a high-potential, high-reward endeavor worthy of focused research. The potential to create a superior, multifunctional cancer therapeutic aligns perfectly with the goals of personalized medicine. While the obstacles are real, they are not insurmountable. Researchers should vigorously pursue this path, as success here could yield a powerful new weapon in the oncological arsenal, fundamentally changing treatment paradigms for some of the most challenging cancers.

## Figures and Tables

**Table 2:** Physical and biological characteristics of α, β particles, and Auger electrons. Data from [29].

	Alpha Particle	Beta Particle	Auger Electron
Type of particles	^4^He nucleus	Energetic electron	Low energy electron; electron capture and/or internal conversion
Particle energy	4–9 MeV	50–2300 keV	25–80 keV
Particle path length	40–100 μm	0.05–12 mm	Nanometers
Linear energy transfer	~80 keV/μm	~0.2 keV/μm	4–26 keV/μm
Hypoxic tumors	Effective	Less effective	Effective
Toxicity	Effective in creating double-strand breaks in DNA	High dose rates (tumor survival rates close to linear exponential). Low dose rates (single-strand breaks), repairable with shouldering the dose–response curve	Potential creation of double-strand breaks DNA and cell membrane
Bystander effect/crossfire	Yes/low	Yes	Yes
Tumor size	Micro/small	Higher volume solid tumor	Micro

**Table 3:** Current ^177^Lu-labeled bifunctional radioligand-based radiopharmaceuticals and their treatment conditions. Data from [51].

^177^Lu Bifunctional Radioligand-Based Radiopharmaceuticals	Treatment Conditions
^177^Lu-DOTATATE (Lutathera^®^)	Gastroenteropancreatic neuroendocrine tumors
^177^Lu-PSMA-617 (Pluvicto^®^)	Prostate-specific membrane antigen–positive metastatic castration-resistant prostate cancer
^177^Lu-J591	Prostate-specific membrane antigen–positive metastatic castration-resistant prostate cancer
^177^Lu-DOTATOC	Neuroendocrine tumors
^177^Lu-PSMA-I&T	Prostate-specific membrane antigen–positive metastatic castration-resistant prostate cancer
^177^Lu-DOTA-HH1 (Betalutin)	B-cell non-Hodgkin lymphomas
^177^Lu-PNT2002	Metastatic castration-resistant prostate cancer
^177^Lu-DOTA-ABM-5G	Advanced/metastatic pancreatic ductal adenocarcinoma
^177^Lu-DOTA-EB-TATE	Metastatic neuroendocrine tumors
^177^Lu-CC49	Prostate-specific membrane antigen–positive metastatic castration-resistant prostate cancer
CTT1403	Prostate-specific membrane antigen–positive metastatic castration-resistant prostate cancer
^177^Lu-DOTA-JR11	Neuroendocrine tumors
^177^Lu-NeoB	Gastrin-releasing peptide receptor-positive tumors
^177^Lu-girentuximab	Metastatic clear cell renal cell carcinoma
GD2-SADA:177Lu-DOTA	GD2-positive solid tumors
^177^Lu-FAP-2286	Fibroblast activation protein-positive solid tumors
^177^Lu-Ludotadipep	Metastatic castration-resistant prostate cancer
^177^Lu-rhPSMA-10.1	Prostate-specific membrane antigen–positive metastatic castration-resistant prostate cancer
^177^Lu-DOTA.SA.FAPi	Solid tumors
^177^Lu-DOTAGA.(SA.FAPi)2	Solid tumors
^177^Lu-FAPi-46	Solid tumors
^177^Lu-trastuzumab	Breast cancer
^177^Lu-brentuximab	Hypoxic tumors
^177^Lu-PP-F11N	Medullary thyroid cancer

**Table 4:** Structures of ^177^Lu chelators employed in the development of bifunctional radioligands [20,47].

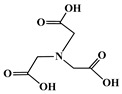	Nitrilotriacetic acid (NTA)
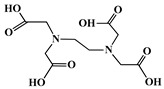	Ethylenediaminetetraacetic acid (EDTA)
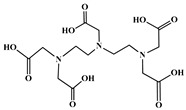	Diethylenetriaminepentaacetic acid (DTPA)
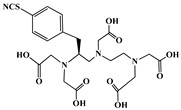	p-SCN-Bn-1B-DTPA
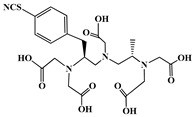	p-SCN-Bn-1B4M-DTPA
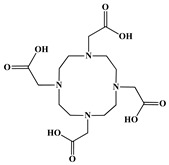	1,4,7,10-Tetraazacyclododecane-1,4,7,10-tetraacetic acid (DOTA)
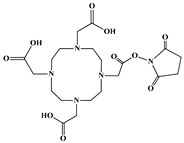	DOTA-NHS-ester
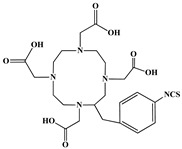	p-SCN-Bn-DOTA
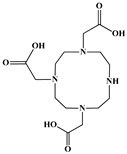	1,4,7,10-Tetraazacyclododecane-1,4,7-triacetic acid (DO3A)
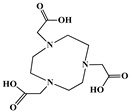	1,4,7-Triazacyclononane-1,4,7-triacetic acid (NOTA)
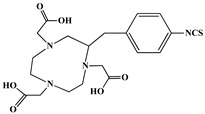	p-SCN-Bn-NOTA
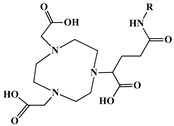	(NOTA (1,4,7-triazacyclononane-1,4,7-triacetic acid) + GA (glutaric acid)) NOTAGA (R = NHS-ester, amide)
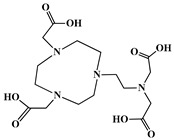	{4-[2-(bis-carboxymethylamino)-ethyl]-7- carboxymethyl-[1,4,7]-triazonan-1-yl}-acetic acid (NETA)
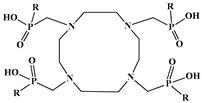	1,4,7,10-tetraazacyclodecane-1,4,7,10- tetra(R)ester phosphinic acid (DOTRP)
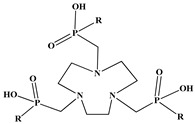	1,4,7-teriazacyclononane-1,4,7-tri(R)ester phosphinic acid (NORP)
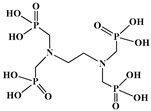	Ethylenediamine-tetra(methylene-phosphonic acid), (EDTMP)
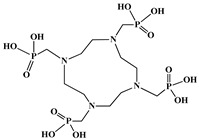	1,4,7,10-tetraazacyclododecane-1,4,7,10- tetramethylene-phosphonic acid (DOTMP)

**Table 5:** Log stability constants of Lu(III) complexes with well-known chelating agents. Data from [19,47].

Ion	NTA	EDTA	DTPA	NOTA	DOTA	DO3A	DOTMP
Lu(III)	12.49	19.83	22.44	15.3	25.4	23.0	29.6

**Table 6:** Global ^177^Lu market research report 2025. Data from [73,74].

Metrics	Details
Accounted market size in the year 2025	US$ 2.73 billion
Forecasted market size in 2032	US$ 10.84 billion
Compound annual growth rate from 2025 to 2032	21.8%
Production by type	Non-carrier-added Carrier-added
Production by application	Nuclear Therapy Others
Production by region	North America Europe Australia South Africa
Consumption by region	North America (United States, Canada) Europe (Germany, France, UK, Italy, Russia) Asia-Pacific (China, Japan, South Korea, Taiwan) Southeast Asia (India) Latin America (Mexico, Brazil)
Production by company	Advanced Accelerator Applications (Novartis) Saint-Genis-Pouilly, France Eckert & Ziegler Strahlen, Berlin, Germany SHINE Technologies, Janesville, WI, USA ANSTO, Sydney and Melbourne, Australia NTP Radioisotopes, Pelindaba, South Africa

**Table 7:** Active, terminated, or completed recent clinical trials on ^177^Lu-based radiopharmaceuticals. Data from [75].

Study Title	Status	Conditions	Interventions	Study Type	Adverse Events and Outcomes
Evaluation of safety and dosimetry of Lutathera in adolescent patients with gastroenteropancreatic neuroendocrine tumors (GEP-NETs) and pheochromocytomas and paragangliomas (PPGLs)	Active	GEP-NETs PPGLs	Drug: ^177^Lu-Oxodotreotide/dotatate	Interventional	
^177^Lu-PSMA-617 vs. androgen receptor-directed therapy in the treatment of progressive metastatic castrate resistant prostate cancer	Active	Prostatic neoplasms	Drug: 177Lu-PSMA-617 Drug: ^68^Ga-PSMA-11 Drug: ARDT		
Telotristat with Lutathera in neuroendocrine tumors (NETs)	Terminated	NETs	Drug: Telotristat (Low dose) Drug: Telotristat (High dose)		Zero adverse events reported. However, only 1 participant was enrolled in this study. Based on the low enrolment number, no data is reported here in order to protect and maintain participant privacy/confidentiality.
Post-authorization safety study of LysaKare^®^ in adult GEP-NET patients	Completed	GEP-NETs	Drug: arginine/lysine to protect the kidneys from radiation damage during cancer treatment with ^177^Lu-Oxodotreotide.		All participants enrolled in the study received one dose of arginine/lysine solution administered intravenously over a 4 h period. Zero serious adverse events were reported. There is an agreement between principal investigators (PIs) and the sponsor (or its agents) that restricts the PI’s rights to discuss or publish trial results after the trial’s completion.
^177^Lu-DTPA-Omburtamab radioimmunotherapy for recurrent or refractory medulloblastoma	Terminated	Medulloblastoma in childhood	Drug: ^177^Lu-DTPA-Omburtamab		From 1st dose to 5 weeks after last dose, up to 10 weeks (2 cycles). Partial seizures were reported as serious adverse events for all participants.
Evaluation of efficacy and safety of Lutathera in patients with grade 2 and grade 3 advanced GEP-NET	Active	GEP-NETs	Drug: Lutathera Drug: 30 mg Octreotide long-acting repeatable (LAR) Drug: 2.5% Lysine-Arginine sterile amino acid solution Drug: High dose 60 mg Octreotide LAR		
A trial of CTT1403 for metastatic castration resistant prostate cancer (mCRPC)	Completed	Prostate cancer	Drug: CTT1403 Drug: CTT1057 Drug: ^68^Ga-PSMA-11		Information on adverse events was collected throughout the course of the study and up to 6 months after the last administered dose of the study drug, up to a total of 7 months. The highest administered dose of CTT1403 was 9.0 GBq. There is an agreement between PIs and the sponsor (or its agents) that restricts the PI’s rights to discuss or publish trial results after the trial’s completion.
Evaluation of safety and efficacy of Betalutin and Rituximab in patients with follicular lymphoma	Completed	Non-Hodgkin lymphoma Follicular lymphoma Relapsed follicular lymphoma	Drug: 10 MBq/kg Betalutin Drug: 15 MBq/kg Betalutin		All adverse events were collected from the time of informed consent until 4 weeks after the last rituximab dose, up to 25 months. Thereafter, treatment-related adverse events were collected up to 36 months for the first two participants only. Erysipelas, bronchitis viral, thrombocytopenia, palpitations, and other gastrointestinal and general disorders were reported as serious and less serious adverse events.
^177^Lu-PSMA-617 and Pembrolizumab in treating patients with mCRPC	Completed	Castration levels of testosterone Castration-resistant prostate carcinoma Metastatic prostate carcinoma Prostate adenocarcinoma Stage IV porostate cancer Stage IVA prostate cancer Stage IVB prostate cancer	Drug: ^177^Lu-PSMA-617 Biological: Pembrolizumab		The additional efficacy endpoints, including median duration of response, PSA50 response rate, rPFS rate at 6 months, median PSA progression-free survival, median OS, and median time to SSRE, were planned to be analyzed in the overall study cohort (*n* = 43). Post hoc determination of efficacy outcomes for schedules 1,2, 3 analyzed separately is precluded by the limited sample size of patients enrolled on schedules 2 and 3 (*n* = 6 each). More information can be found in [76].
Evaluation of safety, tolerability, biodistribution, and anti-tumor activity of ^177^Lu-OPS201 with companion imaging ^68^Ga-OPS202 PET/CT in previously treated subjects with locally advanced or metastatic cancers expressing Somatostatin Receptor 2 (SSTR2)	Terminated	Small cell lung cancer and breast cancer	Drug: Satoreotide tetraxetan Drug: Satoreotide trizoxetan		This study was terminated early due to unsuccessful screening and not due to subjects’ safety. No outcomes measures were evaluated as no subjects received the therapeutic ^177^Lu-satoreotide tetraxetan. There is an agreement between PIs and the sponsor (or its agents) that restricts the PI’s rights to discuss or publish trial results after the trial’s completion.
^177^Lu-J591 and ^177^Lu-PSMA-617 combination for mCRPC	Terminated	Prostate cancer	Drug: ^177^Lu-PSMA-617 Drug: ^177^Lu-J591 Drug: ^68^Ga-PSMA-HBED-CC		The study was terminated early due to sponsor withdrawal (PSMA-617 no longer available for purchase). More information can be found in [77].
Evaluation of safety and activity (including distribution) of ^177^Lu-3BP-227 in subjects with solid tumors expressing Neurotensin Receptor Type 1.	Terminated	Pancreatic ductal adenocarcinoma Colorectal cancer Gastric cancer Squamous cell carcinoma of the head and neck Bone cancer Advanced cancer Recurrent disease Metastatic tumors	Drug: ^177^Lu-3BP-227 (also called ^177^Lu-IPN01087)		The sponsor terminated the study early and phase 1 dose expansion and phase 2 were not started. The decision to terminate the study was not due to any safety or tolerability concern, or any event associated with the use of ^177^Lu-3BP-227.
Study of ^177^Lu-PSMA-617 in mCRPC	Completed	Prostate cancer	Drug: ^177^Lu-PSMA-617 Other: Best supportive/best standard of care		There is an agreement between PIs and the sponsor (or its agents) that restricts the PI’s rights to discuss or publish trial results after the trial’s completion. However, the following publications are provided voluntarily by the person who enters information about the study and are about the study results [78,79,80,81,82,83,84,85,86,87,88].
Nivolumab and ^177^Lu-DOTA0-Tyr3-Octreotate for patients with extensive-stage small cell lung cancer	Completed	Small cell lung cancer Small cell lung cancer extensive stage	Drug: Nivolumab Radiation: ^177^Lu-DOTA0-Tyr3-Octreotate		There is an agreement between PIs and the sponsor (or its agents) that restricts the PI’s rights to discuss or publish trial results after the trial’s completion. More information can be found in [89].
Phase I dose-escalation study of fractionated ^177^Lu-PSMA-617 for progressive mCRPC	Active	Prostate cancer	Drug: ^177^Lu-PSMA-617 Drug: ^68^Ga-PSMA-HBED-CC		
^177^Lu prostate-specific membrane antigen (PSMA)-directed endoradiotherapy	Terminated	mCRPC	Drug: ^177^Lu-PSMA-617		The study began on 5 July 2017as an Investigator Initiated Trial and sponsorship was transferred to Endocyte on 1 June 2018. Recruitment was stopped before the target sample size was achieved based on strategic considerations.
Study of Betalutin for treatment of relapsed or refractory Non-Hodgkin lymphoma (LYMRIT-37-05)	Completed	Relapsed, diffuse large B-cell lymphoma Refractory diffuse large B-cell lymphoma	Drug: Betalutin		There is an agreement between PIs and the sponsor (or its agents) that restricts the PI’s rights to discuss or publish trial results after the trial’s completion. More information can be found in [90,91].
Evaluation of safety and preliminary efficacy of ^177^Lu-OPS201 in NETs	Terminated	NETs	Drug: Satoreotide tetraxetan Other: Amino acid solution Other: Antiemetic		Due to strategic reasons, the Ipsen management team decided to early terminate the D-FR-01072-00 /OPS-C-001 study. This decision was not due to any safety or tolerability concern, or any event associated with the use of the study drug.
A phase 1/2 study of Betalutin for treatment of relapsed Non-Hodgkin lymphoma	Completed	Non-Hodgkin lymphoma Follicular lymphoma	Drug: 10 MBq/kg Betalutin Drug: 15 MBq/kg Betalutin Drug: 20 MBq/kg Betalutin Drug: 40 mg lilotomab Drug: 100 mg/m^2^ lilotomab Drug: 60 mg/m^2^ lilotomab Drug: Rituximab Drug: 12.5 mBq/kg Betalutin		There is an agreement between PIs and the sponsor (or its agents) that restricts the PI’s rights to discuss or publish trial results after the trial’s completion. More information can be found in [90,91,92,93].
A study comparing treatment with ^177^Lu-DOTA0-Tyr3-Octreotate to Octreotide LAR in patients with inoperable, progressive, SSTR-positive midgut carcinoid tumors	Completed	Carcinoid tumor of the small bowel NETs	Drug: Octreotide LAR Drug: ^177^Lu-DOTA0-Tyr3-Octreotate		There is an agreement between PIs and the sponsor (or its agents) that restricts the PI’s rights to discuss or publish trial results after the trial’s completion. More information can be found in [94,95,96,97].
^177^Lu radiolabeled monoclonal antibody HuJ591 (^177^Lu-J591) and Ketoconazole in patients with prostate cancer	Active	Prostate cancer	Drug: ^177^Lu-J591 Drug: Ketoconazole Drug: Hydrocortisone Drug: ^111^In-J591		
Treatment with radiolabeled monoclonal antibody HuJ591-GS (^177^Lu-J591) in patients with metastatic prostate cancer	Completed	Prostate cancer	Drug: ^177^Lu-J591		These publications are provided voluntarily by the person who enters information about the study and are about the study results [98,99].
Phase I/II dose-escalation study of ^177^Lu-labeled cG250 in patients with advanced renal cancer	Completed	Metastatic renal cell carcinoma	Drug: ^111^In-DOTA-cG250 Drug: ^177^Lu-DOTA-cG250		There is an agreement between PIs and the sponsor (or its agents) that restricts the PI’s rights to discuss or publish trial results after the trial’s completion. More information can be found in [100,101].

## Abbreviations

The following abbreviations are used in this manuscript:

^177^Lu	Lutetium-177
TRT	Targeted radiation therapy
MNPs	Magnetic nanoparticles
NPs	Nanoparticles
MRI	Magnetic resonance imaging
SPECT	Single Photon Emission Computed Tomography
PET	Positron Emission Tomography
ICRP	International Commission on Radiological Protection
Lutathera^®^	[^177^Lu]Lu-DOTATATE
Pluvicto^®^	[^177^Lu]Lu-Vivipotide tetraxetan
PRRT	Peptide receptor radionuclide therapy
CuAAC	Copper(I)-catalyzed azide-alkyne cycloaddition
SPAAC	Strain-promoted azide-alkyne cycloaddition
IEDDA	Inverse-electron-demand Diels–Alder
CBT	2-cyanobenzothiazole
NTA	Nitrilotriacetic acid
EDTA	Ethylenediaminetetraacetic acid
DTPA	Diethylenetriaminepentaacetic acid
DOTA	1,4,7,10-Tetraazacyclododecane-1,4,7,10-tetraacetic acid
DO3A	1,4,7,10-Tetraazacyclododecane-1,4,7-triacetic acid
NOTA	1,4,7-Triazacyclononane-1,4,7-triacetic acid
NOTAGA	(NOTA (1,4,7-triazacyclononane-1,4,7-triacetic acid) + GA (glutaric acid))
NETA	{4-[2-(bis-carboxymethylamino)-ethyl]-7-carboxymethyl-[1,4,7]-triazonan-1-yl}-acetic acid
DOTRP	1,4,7,10-tetraazacyclodecane-1,4,7,10-tetra(R)ester phosphinic acid
NORP	1,4,7-teriazacyclononane-1,4,7-tri(R)esterphosphinic acid
^177^Hf	Hafnium-177
^176^Yb	Ytterbium-176
^176^Lu	Lutetium-176
^169^Yb	Ytterbium-169
^175^Yb	Ytterbium-175
^177^Yb	Ytterbium-177
SA	Specific activity
GEP-NETs	Gastroenteropancreatic neuroendocrine tumors
PPGLs	Pheochromocytomas and paragangliomas
LAR	Long-acting repeatable
SSTR2	Somatostatin Receptor 2
mCRPC	Metastatic castration-resistant prostate cancer
PI(s)	Principal investigator(s)
PSMA	Prostate-specific membrane antigen
PET/CT	Positron emission tomography/Computed tomography
NETs	Neuroendocrine tumors
EPR	Enhanced permeability and retention
MIONPs	Magnetic iron oxide NPs
IONPs	Iron oxide NPs
SPIONPs	Superparamagnetic iron oxide NPs
SPIO	Superparamagnetic iron oxide
Fe_3_O_4_	Magnetite
*γ*-Fe_2_O_3_	Maghemite
PEG	Polyethylene glycol
co-CNCs	Condensed colloidal nanocrystal clusters
MA	Alginate-coated nanocrystallites
MAPEG	PEGylated MA
D_h_	Hydrodynamic diameter
DLS	Dynamic light scattering
ITLC-SG	Instant Thin Layer Chromatography-Silica Gel
NBT	Nanobrachytherapy
DMSA	Meso-1,2-dimercaptosuccinic acid
TEM	Transmission electron microscopy
PDI	Polydispersity index
XRD	X-ray diffraction
FT-IR	Fourier-transform infrared
CA	Citric acid
PAA	Poly(acrylic acid)
SAED	Selected-area electron diffraction
M_S_	Saturation magnetization
EDC	N-ethyl-N′-(3-(dimethylamino)propyl)carbodiimide
NHS	N-hydroxysuccinimide
AA	Alginic acid
DOX	Doxorubicin
BVCZ	Bevacizumab
HPLC	High-performance liquid chromatography
TGA	Thermogravimetric analysis
RP-HPLC	Reverse-phase high-performance liquid chromatography
PBS	Phosphate-buffered saline
HIR	Heat-induced radiolabeling
FH	Feraheme
CMD	Carboxymethyldextran
ICP-MS	Inductively coupled plasma mass spectrometry
SEC	Size exclusion chromatography
LAGMERAL	Ligand Anchoring Group-Mediated Radiolabeling
DP-PEG	Diphosphonate-polyethylene glycol
FBS	Fetal bovine serum
ccDTPA	Cyclohexane-1,4-diyldinitrilo)tetraacetic acid dianhydride
MACS	Magnetic-assorting cell separation
PCS	Photon correlation spectroscopy
PLL	Poly-L-lysine
SAR	Specific absorption rate
Pro	Proline
Trp	Tryptophan
UV-Vis	Ultraviolet-Visible
DCS	Differential centrifugal sedimentation
HA	Hyaluronic acid
DBCO	Dibenzocyclooctyne
^177^Lu-TCL-SPIONPs	^177^Lu-labeled thermally cross-linked SPIONPs
RES	Reticuloendothelial system
VEGF	Vascular Endothelial Growth Factor
NMIBC	Nonmuscle-invasivebladder cancer
MIBC	Muscle-invasive bladder cancer
^177^Lu-MNFs	^177^Lu-labeled magnetic nano-formulations
GMP	Good Manufacturing Practice
RCY	Radiochemical yield
RCP	Radiochemical purity
